# Microencapsulation of Grape Pomace Extracts with Alginate-Based Coatings by Freeze-Drying: Release Kinetics and In Vitro Bioaccessibility Assessment of Phenolic Compounds

**DOI:** 10.3390/gels10060353

**Published:** 2024-05-21

**Authors:** Josipa Martinović, Rita Ambrus, Mirela Planinić, Gordana Šelo, Ana-Marija Klarić, Gabriela Perković, Ana Bucić-Kojić

**Affiliations:** 1 Faculty of Food Technology Osijek, Josip Juraj Strossmayer University of Osijek, HR-31 000 Osijek, Croatia; 2 Faculty of Pharmacy, Institute of Pharmaceutical Technology and Regulatory Affairs, University of Szeged, H-6720 Szeged, Hungary

**Keywords:** grape pomace extracts, encapsulation, freeze-drying, bioaccessibility, phenolic compounds

## Abstract

The phenols from grape pomace have remarkable beneficial effects on health prevention due to their biological activity, but these are often limited by their bioaccessibility in the gastrointestinal tract. Encapsulation could protect the phenolics during digestion and influence the controlled release in such an intestine where their potential absorption occurs. The influence of freeze-drying encapsulation with sodium alginate (SA) and its combination with gum Arabic (SA-GA) and gelatin (SA-GEL) on the encapsulation efficiency (*EE*) of phenol-rich grape pomace extract and the bioaccessibility index (*BI*) of phenolics during simulated digestion in vitro was investigated. The addition of a second coating to SA improved the *EE*, and the highest *EE* was obtained with SA-GEL (97.02–98.30%). The release of phenolics followed Fick’s law of diffusion and the Korsmeyer–Peppas model best fitted the experimental data. The highest *BI* was found for the total phenolics (66.2–123.2%) and individual phenolics (epicatechin gallate 958.9%, gallocatechin gallate 987.3%) using the SA-GEL coating were used. This study shows that freeze-dried encapsulated extracts have the potential to be used for the preparation of various formulations containing natural phenolic compounds with the aim of increasing their bioaccessibility compared to formulations containing non-encapsulated extracts.

## 1. Introduction

Grape pomace, a by-product of the wine industry, is a significant source of phenolic compounds with potential health benefits [1]. These compounds, which are contained in the skins, seeds and stems after pressing the grapes, include phenolic acids, flavonols, flavanols, stilbenes and anthocyanins [2]. Interest in grape pomace phenols is growing due to their antioxidant, anti-inflammatory and cardioprotective properties, which are crucial for the prevention of chronic diseases that are constantly on the rise, such as cancer and cardiovascular diseases [3]. However, their practical application is limited by problems such as poor solubility, stability under environmental conditions and low bioavailability in the human gastrointestinal tract [4]. Encapsulation techniques including freeze-drying, have proven to be a promising approach to mitigate these problems. To further elucidate the application of encapsulation technologies in the context of winemaking by-products, this study delves into the use of freeze-drying techniques for phenolic extracts from grape pomace, a significant waste stream from the wine industry. Encapsulation not only protects these sensitive phenolic compounds from oxidative stress and degradation, but also can improve their solubility and stability, which is critical for effective delivery in the human gastrointestinal tract.

Freeze-drying is a preferred encapsulation method because it preserves the integrity and bioactivity of phenolic compounds by removing water at low temperature and pressure, thereby maintaining the structural stability of the encapsulated product [5]. To improve the effectiveness of this process, various biopolymers such as sodium alginate (SA), gum Arabic (GA) and gelatin (GEL) are used as coating materials. These coatings are selected for their ability to form a gel and their compatibility with the freeze-drying process and are therefore suitable for creating a protective matrix with phenolic compounds. The selection and combination of these coatings are critical as they influence the encapsulation efficiency, protect the active ingredients from oxidative damage and control the release behavior during digestion [6]. An important aspect of the use of encapsulated phenolic compounds is their bioaccessibility and release profile in a simulated gastrointestinal environment, which are usually evaluated by in vitro studies. The release behavior of encapsulated compounds can be mathematically modeled to predict their release and functional efficacy [7,8]. Such modeling is crucial for optimizing encapsulation and release strategies and ensures that the phenolic compounds are available in the right place and at the right time in the digestive tract to maximize their health benefits.

The aim of this study is to analyze the phenolic profiles of different samples of phenol-rich grape pomace extracts and to investigate the effects of different alginate-based coatings used in encapsulation by freeze-drying on the encapsulation efficiency of the phenolic compounds, their release behavior and their bioaccessibility during simulated digestion in vitro.

There are little data in the literature on the application of these natural coatings for freeze-drying, and the novelty of this work is precisely to fill this gap. This comprehensive study will help to promote the sustainable use of waste and knowledge on the better utilization of phenolic compounds from winery waste streams and to understand the release mechanisms and bioavailability of encapsulated phenolic compounds in the human gastrointestinal system. This dual contribution represents a significant advance in the field of food science and demonstrates the desirability of moving from linear to circular waste management.

## 2. Results and Discussion

### 2.1. The Phenolic Compounds Composition of Grape Pomace Extracts

**Figure 1:**
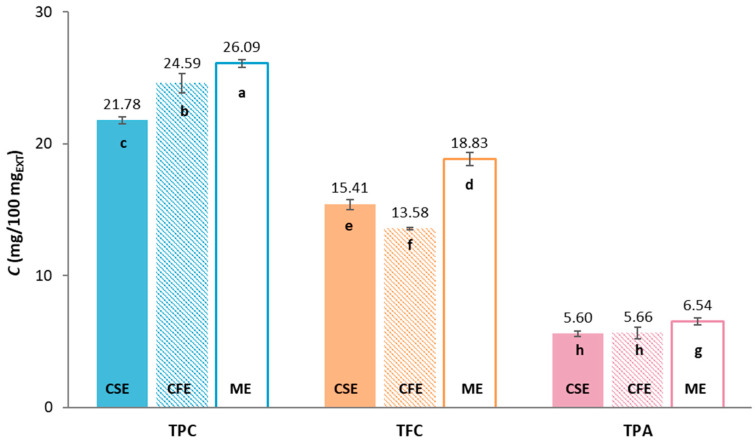
Content of total phenolic compounds (TPC), total flavonoids (TFC) and total extractable proanthocyanidins (TPA) of Cabernet Sauvignon (CSE); Cabernet Franc (CFE) and Merlot (ME) grape pomace extracts (EXT). Different letters stand for statistically significant differences within the individual result groups (TPC, TFC, TPA) (ANOVA, post hoc Duncan’s test at *p* < 0.05).

The total phenolic content (TPC), total flavonoids (TFC) and total extractable proanthocyanidins (TPA) of three different grape pomaces: Cabernet Sauvignon (CSE), Cabernet Franc (CFE) and Merlot (ME), were determined spectrophotometrically, and the results are shown in Figure 1.

Among the groups of compounds tested, TPC is predominant in all tested extracts, ranging from 21.78 to 26.09 mg_GAE_/100 mg_EXT_, followed by TFC (13.58–18.83 mg_CE_/100 mg_EXT_) while TPA has the lowest content, ranging from 5.60 to 6.54 mg/100 mg_EXT_. The highest content of certain phenolic compounds was found in sample ME. It can be seen that the sample has a statistically significant influence on the content of TPC and TFA. For TPA, there was no statistically significant difference in the content of samples CSE and CFE, while the TPA content of ME was statistically significantly different from them. Comparing the results obtained with the available literature data, there is considerable variability due to many factors, including variety, grape pomace composition (including pulp, seeds, grape skins and sometimes stalks and other solid residues after pressing), climatic conditions, agrotechnical conditions, geographical location, winemaking process and extraction methods [1]. For example, Rockenbach et al. [9] analyzed the TPC of CSE and ME obtained from the Videira winery (Brazil, vintage 2008) and found lower contents of TPC compared with this study, namely 7.48 mg_GAE_/100 mg_EXT_ for CSE and 4.62 mg_GAE_/100 mg_EXT_ for ME. Similarly, the studies of Iora et al. [10] included the analysis of CSE and ME varieties grown in the Toledo region (Brazil, vintage 2012), where they recorded a higher content of TPC and TFC in CSE (TPC = 5.10 mg_GAE_/100 mg_EXT_; TFC = 2.98 mg_CE_/100 mg_EXT_) than in ME (TPC = 3.76 mg_GAE_/100 mg_EXT_; TFC = 2.12 mg_CE_/100 mg_EXT_). In addition, Xu et al. [11] investigated the composition of the CFE originated from Orange County (Orange, VA, USA) and determined the content of TPC 15.38 mg_GAE_/100 mg_EXT_ and TFC 9.17 mg_CE_/100 mg_EXT_, while Jin et al. [12] determined 36.1 mg_GAE_/g_db_ for TPC, 16.3 mg_CE_/g_db_ for TFC and 21.2 mg/g_db_ for TPA for CFE from the Crozet region (Crozet, VA, USA).

The profile of the individual phenolic compounds of all grape pomace extracts determined by ultra-high performance liquid chromatography (UHPLC) is shown in Table 1.

Of the 33 phenolic compounds tested in grape pomace extracts, 26 phenolic compounds were identified and quantified in CSE and ME and 27 phenolic compounds in CFE prior to simulated digestion in vitro. These phenolic compounds are divided into five groups: phenolic acids (hydroxybenzoic acid and hydroxycinnamic acid), flavanols, flavonols, stilbenes and anthocyanins.

All extracts contain significant amounts of hydroxybenzoic phenolic acids: ellagic acid (16.32–94.72 mg/100 mg_EXT_), 3,4-dihydroxybenzoic acid (24.09–75.63 mg/100 mg_EXT_), syringic acid (51.89–114.36 mg/100 mg_EXT_) and gallic acid (130.00–207.79 mg/100 mg_EXT_). Gallic acid, which is abundant in various fruits and vegetables, has various biological functions, such as anti-cancer, antimicrobial and antioxidant effects. However, problems such as poor solubility, stability and low bioavailability hinder its therapeutic potential [13]. Syringic acid, known for its antioxidant properties and benefits such as liver protection [14], anti-inflammation [15], antidiabetic [16] and neuroprotection [17], encounters similar limitations in therapeutic efficacy due to its low bioavailability [18]. Similarly, ellagic acid, although offering the same health benefits as gallic acid and syringic acid, encounters limitations due to its solubility and bioavailability, necessitating research into controlled-release formulations in the gastrointestinal tract [19]. 3,4-dihydroxybenzoic acid, on the other hand, shows promising properties, including anti-inflammatory, neuroprotective, antidiabetic and antioxidant effects, suggesting potential therapeutic applications [20]. Although not found in high concentrations in grape pomace, hydroxycinnamic acids—caffeic acid, ferulic acid and coumaric acids—are extremely important due to their numerous beneficial biological effects. They possess antioxidant and antitumor properties and contribute to the prevention of cardiovascular diseases and hypertension [21,22,23], and a diet enriched with hydroxycinnamic acids reduces the risk of Alzheimer’s disease and atherosclerosis [24,25]. Of the hydroxycinnamic acids examined, *o*-coumaric acid was quantified in the highest concentrations in all grape pomace extracts (7.46–19.48 mg/100 mg_EXT_).

The flavanols present in grape skins are mainly in the form of catechins and account for a significant proportion (13–30%) of the total phenolic content in red grapes, while their content is higher in white grape varieties (46–56%) [26]. Table 1 shows that epicatechin (100.71–547.27 mg/100 mg_EXT_) and catechin (240.87–527.59 mg/100 mg_EXT_) together with procyanidin B1 (118.52–317.42 mg/100 mg_EXT_) were among the most abundant phenolic compounds in the analyzed extracts. The flavonol quercetin was another extremely abundant single phenolic compound in the extracts (120.95–214.33 mg/100 mg_EXT_). Flavanols are extremely interesting compounds as their positive influence on cardiovascular health has been demonstrated [27]. Proanthocyanidins show strong anticancer, antimicrobial and chemoprotective activity as well as a strong antioxidant effect [28,29,30,31], and the positive effect of flavonols against osteoporosis has been demonstrated in both in vitro and in vivo studies [32].

Like all other phenolic compounds, stilbenes have an antioxidant effect, but they also contribute to the prevention of cancer and cardiovascular disease and have neuroprotective and anti-inflammatory properties [33]. Resveratrol and its dimer, ε-viniferin, were observed in the extracts studied, with higher levels of ε-viniferin found in CSE (22.75 mg/100 mg_EXT_) and CFE (13.72 mg/100 mg_EXT_); and higher levels of resveratrol in ME (14.16 mg/100 mg_EXT_) (Table 1).

The last group of phenolic compounds investigated were anthocyanins, which are commonly associated with the color of grapes and can be used as natural pigments. However, they also contribute to health through their cardioprotective, antithrombotic, antiatherosclerotic, vasoprotective and anti-inflammatory properties [34]. Table 1 shows large differences in the content of anthocyanins, but also large differences between the contents of the same anthocyanins between the studied extracts. It can be seen that callistephin chloride was identified exclusively in CFE. However, all extracts have in common that the highest concentrations of peonidin-3-*O*-glucoside chloride (7.17–77.75 mg/100 mg_EXT_) and oenin chloride (32.83–794.37 mg/100 mg_EXT_) were quantified (Table 1). Anthocyanins are attributed similar properties to many flavanols and flavones, such as antioxidant, antiviral and anticancer properties [35,36,37].

When considering all extracts, the highest content of the individual compounds was observed in CFE (Table 1), although the highest levels of TPC, TFC and TPA were previously determined in ME (Figure 1).

### 2.2. Encapsulation Efficiency of Total Phenolic Compounds from Grape Pomace Extracts

Encapsulation of phenol-rich grape pomace extracts (CSE, CFE, ME) in different alginate-based coatings was performed by freeze-drying and the encapsulation efficiency was evaluated.

**Figure 2:**
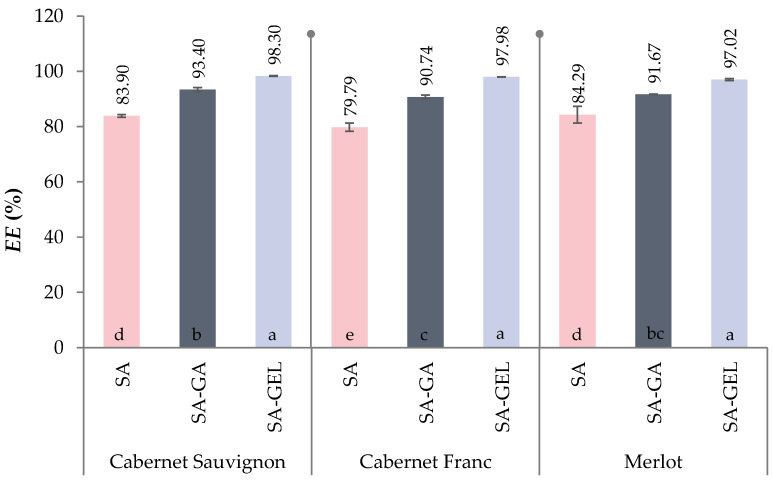
Encapsulation efficiency (*EE*, %) of phenol-rich grape pomace extracts using various coatings (SA—sodium alginate, and combinations of sodium alginate with gum Arabica—SA-GA and with gelatin—SA-GEL). Different letters represent statistically significant differences between results (ANOVA, post hoc Duncan´s test at *p* < 0.05).

The results show that the addition of a second coating to SA increases the encapsulation efficiency (*EE*) of phenolic compounds, which underlines the crucial role of the choice of coating or combination of coatings in improving *EE* (Figure 2). Thus, the *EE* for all samples ranged from 79.79 to 84.29% when SA was used alone, and when SA was used in combination with gum Arabic (SA-GA) or gelatin (SA-GEL), the *EE* increased and ranged from 90.74 to 93.40% and from 97.02 to 98.30%, respectively.

For all encapsulated extracts (CSE, CFE, ME), no statistically significant difference in *EE* was observed when SA-GEL was used as coating and for samples CSE and ME when SA and SA-GA coating was used. The *EE* of sample CFE was statistically different from the other two encapsulated extracts when SA was used as a coating, while there was no difference in *EE* compared to ME when SA-GA was used as a coating. The inclusion of GA or GEL in the encapsulation matrix significantly increases the *EE* due to their protein content, which promotes forming both hydrophobic and hydrogen bonds with the phenolic compounds contained in the grape pomace extracts. These interactions are further enhanced by the ability of the protein to bond with the free carboxyl groups of the polymers, as shown by Li et al. [38] and Jyothi et al. [39].

The results of this study are consistent with the existing literature highlighting the influence of coating(s) on the encapsulation outcome and suggest that the synergistic effects of combining SA with GA or GEL can enhance the protective matrix around the phenolic compounds, leading to higher *EE* regardless of encapsulation methods. Martinović et al. [40] also found that using the ionic gelation method and these two coating combinations significantly improved *EE*, which was 52.62% and 69.27% for the SA-GA and SA-GEL, respectively. The emulsifying and gelling properties of GA [41] and the ability of GEL to improve mechanical strength and barrier properties [42] likely contribute to this improvement by ensuring a more efficient encapsulation process and reducing the loss of phenolic compounds during freeze-drying. These advances in encapsulation technology not only pave the way for improving the stability and bioavailability of bioactive compounds from extracts, but also have significant implications for their application in food and dietary supplements, where optimal *EE* is critical for achieving the desired health benefits and shelf life of the product.

### 2.3. Physicochemical Characterization of Microencapsulated Powder

The product of encapsulation of phenol-rich grape pomace with various alginate- based coatings by freeze-drying was a microencapsulated powder that was subjected to physicochemical characterization, including morphology studies, X-ray powder diffraction (XRPD) and differential scanning calorimetry (DSC) analysis.

#### 2.3.1. Morphology of Microencapsulated Powder

**Figure 3:**
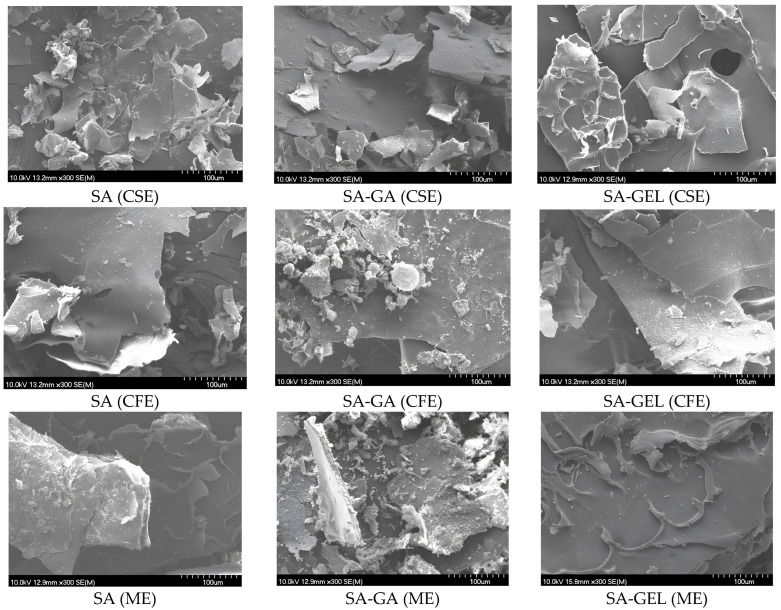
SEM image of microencapsulated powder containing grape pomace extracts (Cabernet Sauvignon (CSE), Cabernet Franc (CFE) and Merlot (ME)) prepared with sodium alginate (SA); SA with gum Arabic (SA-GA) and SA with gelatin (SA-GEL) by freeze-drying and their outer layer at the scale of 100 µm.

The influence of the coatings used on the morphology of the microencapsulated powder of various grape pomace extracts produced by freeze-drying was investigated using a scanning electron microscope (SEM), and the images obtained are shown in Figure 3. The microencapsulated powder obtained exhibited a distinct surface morphology characterized by a fissured, plate-like appearance and a texture reminiscent of sawdust (Figure 3). This morphology is consistent with observations made in several studies in this field [43,44] and highlights a consistent pattern in freeze-dried microencapsulated powder.

In addition to these features, the microencapsulated powders also exhibited areas of smooth surfaces, contributing to a diverse morphological profile. Such physical characteristics of microencapsulated powders are indicative of a high degree of brittleness, a property that allows them to be easily broken into finer particles [45]. This brittleness is a crucial factor for the handling, storage, and application possibilities of the powders, as it may affect the solubility, bioavailability and release mechanisms of the encapsulated compounds.

#### 2.3.2. X-Ray Powder Diffraction and Differential Scanning Calorimetry Analysis

**Figure 4:**
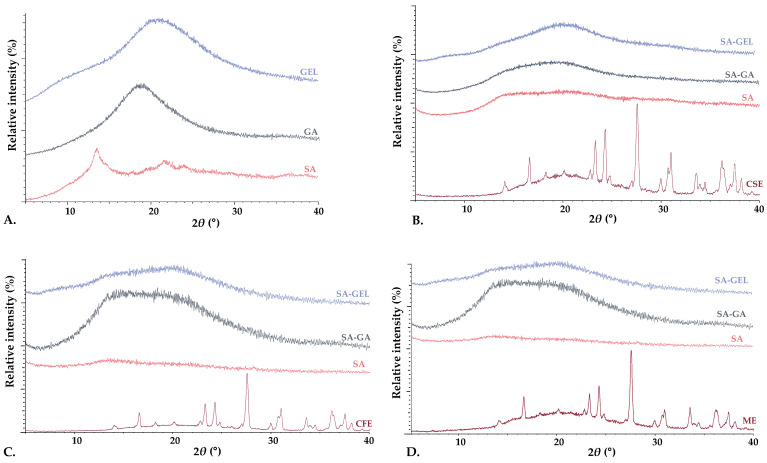
X-ray powder diffractograms of (**A**) pure coatings (sodium alginate—SA, gum Arabic—GA, gelatin—GEL), and microencapsulated powders containing grape pomace phenol-rich extracts of different varieties: (**B**) Cabernet Sauvignon—CSE, (**C**) Cabernet Franc—CFE, (**D**) Merlot—ME, prepared using various coatings (SA, combinations SA-GA and SA-GEL).

XRPD and DSC analyses were used to examine the crystalline and amorphous states of the phenol-rich grape pomace extracts, coatings used and produced microencapsulated powders. XRPD analysis revealed that the coatings were amorphous in structure, characterized by the absence of sharp diffraction peaks (Figure 4A). In contrast, the phenol-rich grape pomace extracts showed distinct sharp peaks in the XRPD spectra, indicating a highly crystalline structure (Figure 4B–D). It is noteworthy that freeze-drying appeared to cause a change in the crystalline structure of the extracts, leading primarily to amorphization. This change to an amorphous state during freeze-drying has been confirmed in numerous studies [46,47] and underlines its importance in improving the solubility and bioaccessibility of the encapsulated compounds.

**Figure 5:**
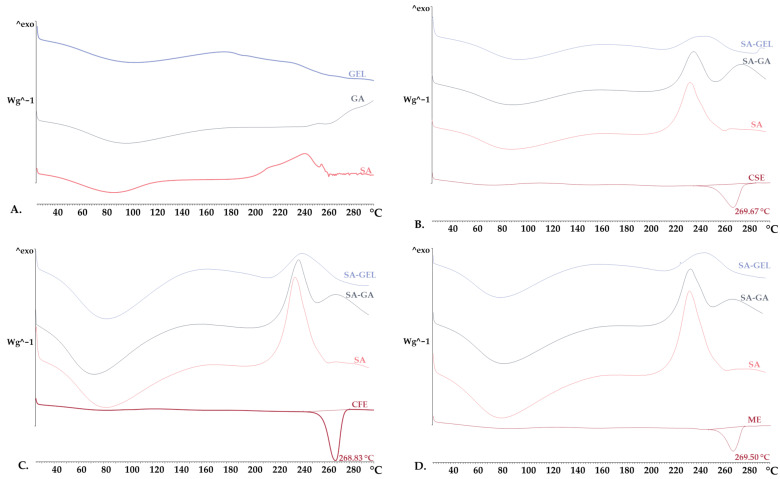
Differential scanning calorimetry thermograms of (**A**) pure coatings (sodium alginate—SA, gum Arabic—GA, gelatin—GEL), and freeze-dried microencapsulated powders containing grape pomace phenol-rich extracts of different varieties: (**B**) Cabernet Sauvignon—CSE, (**C**) Cabernet Franc—CFE, (**D**) Merlot—ME, prepared using various coatings (SA, combinations SA-GA and SA-GEL).

XRPD results were further substantiated by a DSC analysis. The DSC thermograms showed broad peaks in both the pure coatings and the microencapsulated powders, indicating a loss of water during thermal analysis (Figure 5). This is a characteristic feature of materials with amorphous or semi-crystalline structures, where the broad peak is typically associated with the evaporation of free water in the sample [48,49]. On the other hand, the grape pomace extracts exhibited distinct endothermic peaks within a narrow temperature range of 268.83 to 269.67 °C (Figure 5B–D). These sharp transitions are characteristic of the melting point of crystalline extracts.

### 2.4. In Vitro Release of Total Phenolic Compounds from Microencapsulated Powders

**Figure 6:**
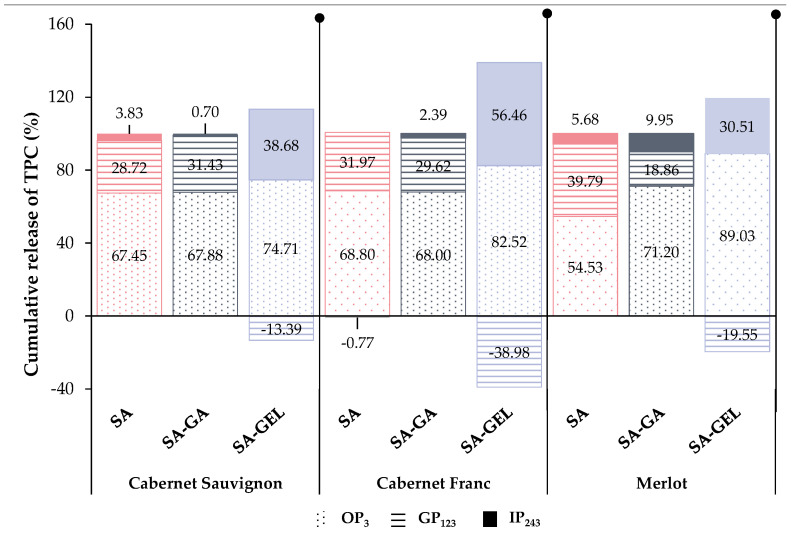
The percentage of cumulative release of total phenolic compounds (TPC) for endpoints of each gastrointestinal phase: OP—oral phase (OP_3_), GP—gastric phase (GP_123_), IP—intestinal phase (IP_243_) from microencapsulated powders containing Cabernet Sauvignon, Cabernet Franc and Merlot grape pomace extracts prepared with sodium alginate (SA), sodium alginate with gum Arabic (SA-GA) and sodium alginate with gelatin (SA-GEL).

The release of TPC from microencapsulated powders containing grape pomace extracts was performed in three phases: oral (OP), gastric (GP) and intestinal phase (IP), using electrolyte solutions without enzymes simulating conditions in the upper human digestive tract (Figure S1 and Figure 6). Afterwards, the following mathematical models were used to describe the kinetics of TPC release from microencapsulated powders: the first-order model, the Higuchi model, the Korsmeyer–Peppas model and the Hixson–Crowell model(Figure S2, Table 2).

The cumulative TPC release profile of CSE-SA microencapsulated powders shows a gradual increase from the beginning of OP (27.41 mg_GAE_/g_P_) to the end of GP (39.08 mg_GAE_/g_P_), with a smaller subsequent increase at the end of IP (40.64 mg_GAE_/g_P_) (Figure S1A). Nevertheless, fluctuations in cumulative TPC release were observed during GP and IP. In contrast to CSE-SA, CFE-SA microencapsulated powders showed a more pronounced cumulative release of TPC in GP, peaking at 50.60 mg_GAE_/g_P_ at 23rd min of in vitro release, followed by a decrease and a slight increase in the cumulative release of TPC in IP at the 163rd min (50.93 mg_GAE_/g_P_) (Figure S1B). ME-SA microencapsulated powders show a similar trend to CSE-SA powders, with a gradual increase until the end of GP (50.25 mg_GAE_/g_P_), followed by a sharp increase in cumulative release of TPC in IP and then a relatively stable release until the end of IP (Figure S1C).

Figure 6 shows the percentage of cumulative TPC released in each phase relative to the total percentage of TPC released during 243 min digestion. Overall, SA as a coating provided a stable cumulative release of TPC in OP (54.53–68.80%) and GP (28.72–39.79%), with a limited release in IP (3.83–5.68%) (Figure 6), indicating that SA may protect TPC in the early stages of digestion, but is not an ideal coating for its release in intestines as the preferred site of absorption when freeze-drying was used for encapsulation. Negative values of cumulative release in IP are visible for CFE-SA powders (−0.77%) (Figure 6), which is a consequence of the release of higher concentrations of TPC at the end of GP than in IP, as shown in Figure S1B.

CSE-SA-GA and CFE-SA-GA microencapsulated powders show a relatively similar pattern of cumulative TPC release. After OP, a uniform release without large differences in TPC content is seen in GP, and at the end of GP, there is an increase in TPC release (32.30–41.08 mg_GAE_/g_P_). The transition to IP leads to a further increase in cumulative release, but also to a rapid decrease in both samples (Figure S1A,B). From the ME-SA-GA microencapsulated powders, a higher content of TPC (32.65 mg_GAE_/g_P_) is already released in the OP than in the two other SA-GA microencapsulated powders, but also compared to the ME-SA and ME-SA-GEL powders (Figure S1). During the GP, fluctuations in the release of TPC are visible, and in the IP, there are no significant changes in the cumulative release (Figure S1C). In Figure 6, it can be observed that the all SA-GA microencapsulated powders show a similar trend in cumulative TPC release as the SA microencapsulated powders, that is, OP < GP < IP with 67.88–71.20% of TPC released in OP, then 18.86–31.43% in GP and 0.70 to 9.95% in IP.

CSE-SA-GEL microencapsulated powders show minimal release during OP, followed by a slight increase in GP and then a decrease in cumulative TPC release. However, upon transition to IP, a significant increase in release and a stable trend during this phase was observed (Figure S1A). A similar trend was observed for CFE-SA-GEL and ME-SA-GEL microencapsulated powders, with the exception in the case of CFE-SA-GEL powders after OP when the cumulative release of TPC showed a decrease during GP (Figure S1B,C). All microencapsulated powders prepared with the SA-GEL coating combination showed negative values of percentage of cumulative TPC release in GP ranging from −13.39 to 39.98%, followed by excellent release in IP (30.51–56.46%) (Figure 6). This is indicative of the results previously seen in Figure S1A–C, where lower concentrations of TPC are released in GP than in IP, and at the same time, the fact that the SA-GEL combination provides the most desirable release profile, i.e., the best protection during the gastric phase and a greater and gradual release of TPC in the intestinal phase, which could potentially allow for better absorption of TPC in the intestinal phase. A similar release profile was also observed after ionic gelation of grape pomace extract using SA-GEL [40] related to the fact that the microbeads swell better at intestinal pH, which allows a lower diffusion of TPC in GP and a higher diffusion in IP [50].

The release kinetics of encapsulated TPC are described by mathematical models shown in Supplementary Materials (Figure S2). The model parameters and statistical criteria used to evaluate the success of the approximation of the experimental data are listed in Table 2. The tested models—the first-order model, the Higuchi model, and the Hixson–Crowell model—showed negative values of the adjusted coefficient of determination (R^2^_adj_) for most of the tested microencapsulated powders, indicating a poor approximation by these models to the experimental data (Table 2).

In contrast, the Korsmeyer–Peppas model showed high R^2^_adj_ values for all three microencapsulated powders prepared with SA (0.889–0.963) and SA-GA (0.873–0.951) coatings, suggesting that this model well represents the experimentally obtained data for the release kinetics of TPC under the conditions tested. For the SA-GEL microencapsulated powders, the Korsmeyer–Peppas model showed slightly lower R^2^_adj_ values for samples CSE and CFE (0.462–0.611), while it is significant that the parameters of this model could not be approximated for the ME-SA-GEL microencapsulated powders (Table 2).

Values of the parameter *n* for SA (0.049–0.084), SA-GA (0.050–0.078) and SA-GEL (0.014–0.093) microencapsulated powders of all samples, which are below 0.45 (Table 2), confirm that the release of TPC from these powders obtained by freeze-drying is primarily governed by Fick’s law of diffusion. The large difference in the range of the parameter *n* in the case of SA-GEL powders indicates more pronounced diffusion, where the movement of phenolic compounds through the encapsulated matrix is largely dependent on the concentration gradient [51]. The presence of gelatin, which is known for its gel-forming properties, could result in the encapsulated network being more uniform or dense, thereby affecting the diffusion of the phenolic compounds [37,52].

The values of the *k*_KP_ parameter for microencapsulated powders prepared with SA were 61.468–77.047, for SA-GA, 64.742–72.207 and 48.962–75.257 for SA-GEL powders (Table 2). These values indicate that the addition of gum Arabic or gelatin influences the properties of the produced microencapsulated powders and thus modulates the release rate of the phenolic compounds. Microencapsulated powders containing gelatin exhibit the widest range of *k*_KP_ values, indicating a potentially greater influence of gelatin on modifying the release rate, possibly due to its interaction with the phenolic compounds or its effect on the structure of the encapsulation matrix [52,53].

### 2.5. In Vitro Simulated Digestion and Bioaccessibility Index of Phenolic Compounds

**Figure 7:**
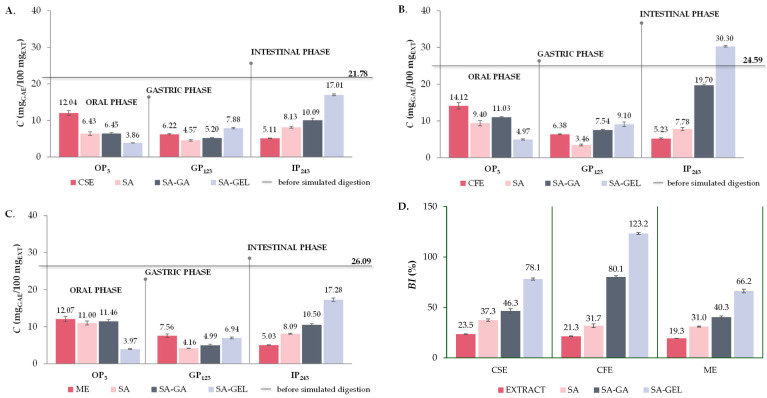
Total phenolic compound content (TPC) before simulated digestion in vitro (–) and at the end of oral (OP_3_), gastric (GP_123_) and intestinal phase (IP_243_) of in vitro simulated digestion of grape pomace phenol-rich extracts (Cabernet sauvignon (CSE)—(**A**), Cabernet Franc (CFE)—(**B**) and Merlot (ME)—(**C**)) and microencapsulated powders containing extracts prepared with sodium alginate (SA), combination of SA with gum Arabic (SA-GA) and combination SA with gelatin (SA-GEL); and the bioaccessibility index (*BI*) of TPC after IP for the tested samples—(**D**).

**Figure 8:**
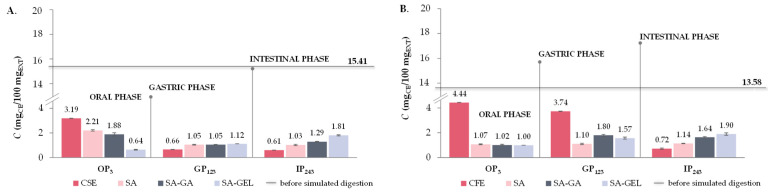

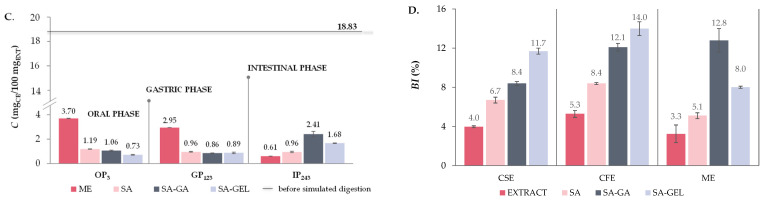
Total flavonoid content (TFC) before simulated digestion in vitro (–) and at the end of oral (OP_3_), gastric (GP_123_) and intestinal phase (IP_243_) of in vitro simulated digestion of grape pomace phenol-rich extracts (Cabernet sauvignon (CSE)—(**A**), Cabernet Franc (CFE)—(**B**) and Merlot (ME)—(**C**)) and microencapsulated powders containing extracts prepared with sodium alginate (SA), combination of SA with gum Arabic (SA-GA) and combination SA with gelatin (SA-GEL); and the bioaccessibility index (BI) of TPC after IP for the tested samples—(**D**).

**Figure 9:**
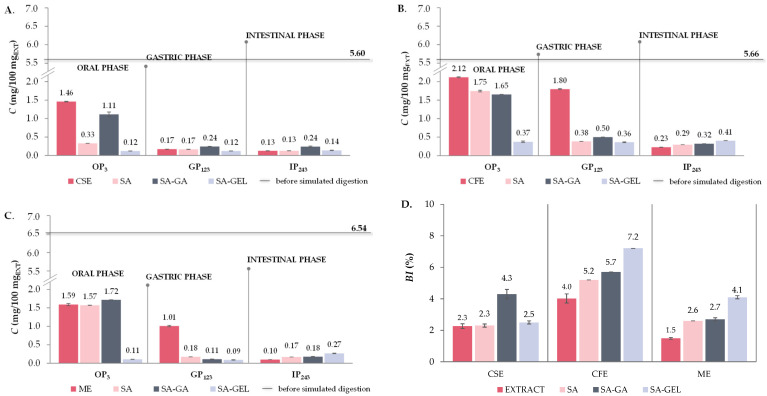
Total extractable proanthocyanidin content (TPA) before simulated digestion in vitro (–) and at the end of oral (OP_3_), gastric (GP_123_) and intestinal phase (IP_243_) of in vitro simulated digestion of grape pomace phenol-rich extracts (Cabernet sauvignon (CSE)—(**A**), Cabernet Franc (CFE)—(**B**) and Merlot (ME)—(**C**)) and microencapsulated powders containing extracts prepared with sodium alginate (SA), combination of SA with gum Arabic (SA-GA) and combination SA with gelatin (SA-GEL); and the bioaccessibility index (BI) of TPC after IP for the tested samples—(**D**).

Phenol-rich grape pomace extracts (CSE; CFE; ME) and microencapsulated powders containing these grape pomace extracts prepared with different coatings (SA; SA-GA; SA-GEL) were subjected to simulated in vitro digestion of the upper digestive tract using digestive enzymes—pepsin, pancreatin and bile extract [54]. The simulated digestion was performed in the OP, GP and IP phases in order to assess the bioaccessibility of phenolic compounds. Bioaccessibility is the proportion of the active ingredient that is released from food into the digestive tract and can potentially be absorbed or is bioavailable and it is the key factor on the bioactivity of phenolic compounds [55,56]. During the execution of the simulated digestion at the end of each phase (OP_3_, GP_123_ and IP_243_; the number in the index indicates the duration of digestion, calculated from the beginning of digestion), samples were obtained in which the concentrations of released TPC, TFC and TPA as well as individual phenolic compounds (phenolic acids, stilbenes, flavanol, flavonol and anthocyanins) were measured for the purpose of testing the influence of SA, SA-GA and SA-GEL coatings on the release profile and bioaccessibility index (*BI*) of the phenolic compounds. The results for TPC, TFC and TPA are presented as mass fractions of cumulatively released phenolic compounds (mg phenolic compound per 100 mg of extract) in each observed digestion phase and compared with the data obtained for phenol-rich grape pomace extracts (Figure 7, Figure 8 and Figure 9), while data for individual phenolic acid are presented as µg/100 mg of extract (Tables S1–S3).

#### 2.5.1. Total Phenolic Compounds, Total Flavonoid Compounds and Total Extractable Proanthocyanidins

The mass fractions of cumulatively released TPC, TFC and TPA from grape pomace extracts and microencapsulated powders containing grape pomace extracts in the digestive fluids at the end of OP, GP and IP and *BI* for the mentioned samples after complete simulated digestion are shown in Figure 7, Figure 8 and Figure 9.

For all extract samples, the cumulative release of TPC is highest during OP (12.04–14.12 mg_GAE_/100 mg_EXT_) (Figure 7A–C), which is to be expected due to the initial exposure to the digestive process and the accelerated diffusion occurring at the beginning. The released TPC then decreases significantly in GP (0.5–0.6-fold) and further decreases in IP (0.7–0.8-fold) compared to GP (Figure 7A–C). This trend suggests that the phenolic compounds are degraded before they reach the intestinal phase, which is not desirable as the goal is a greater release in IP [4]. Microencapsulated powders prepared with SA follow the same trend of TPC release, with a significant decrease in cumulative TPC release in GP (0.4–0.7-fold) compared to OP and then an increase in IP (1.8–2.2-fold) related to GP (Figure 7A–C). Although these results suggest that SA can provide some protection for TPC when freeze drying was used for encapsulation, it cannot completely prevent the degradation of phenolic compounds before they reach the IP.

The TPC release from all SA-GA microencapsulated powders shows a slight decrease from OP to GP (0.4–0.8-fold) and then a significant increase in IP compared to GP (1.9–2.6-fold), especially for encapsulated CFE (Figure 7A–C). This trend suggests that the combination of SA-GA has a protective effect in the first stages of digestion and allows a more significant release of TPC in the IP, which is favorable for the potential absorption of these compounds in IP. The trend of TPC release from SA-GEL microencapsulated powders follows the same trend for all extracts, i.e., TPC release increases as simulated digestion progresses and it is most pronounced in the IP for all encapsulated extracts (Figure 7A–C), with the highest values reported for encapsulated CFE (30.30 mg_GAE_/100 mg_EXT_). This trend of TPC release indicates an excellent protective effect of the SA-GEL coating combination during OP and GP. In line with the release results, the highest *BI* values for TPC were also obtained after simulated digestion of the SA-GEL microencapsulated powders and were in the range of 66.2–123.2% (Figure 7D). The lowest values of *BI* values of TPC were calculated for extracts in the range of 19.3–23.5%, while encapsulation with the freeze-drying method using SA and SA-GA resulted in increases in these values, in the range of 31.0–37.3% and 40.3–80.1%, respectively (Figure 7D).

Like TPC, all extracts show higher TFC release during OP (3.19–4.44 mg_CE_/100 mg_EXT_), whereas TFC release decreases significantly in GP (0.2–0.8-fold) and additionally in the IP (0.2–0.9-fold) (Figure 8A–C). When SA was used for encapsulation of CSE and ME, a similar trend was observed as for the extracts; however, the decrease in release during simulated digestion from OP to IP was less pronounced, indicating the protective effect of SA (Figure 8A,C). Otherwise, for the CFE-SA microencapsulated powders, there is a visible increase in TFC release 1.0-fold in both GP related to OP and IP related to GP (Figure 8B). The TFC release from microencapsulated CSE and ME powders with SA-GA shows a decrease from OP to GP (0.6–0.8-fold) and then a marked increase in IP compared to GP (1.2–2.8-fold) (Figure 8A,C), while the microencapsulated CFE with SA-GA shows a 1.8-fold increase in TFC release in GP and a 0.9-fold decrease in IP (Figure 8B). The TFC release trend observed with microencapsulated CSE and ME suggests that the combination of SA-GA has a protective effect in the initial phases and allows significant release in the IP, which is favorable for the potential absorption of phenolic compounds in IP. The SA-GEL coating combination also shows its effectiveness by achieving the highest release of TFC in the IP (1.68–1.90 mg_CE_/100 mg_EXT_) (Figure 8A–C). According to the release results, *BI* values for TFC determined after simulated digestion of grape pomace extracts ranged from 3.3 to 5.3%, and with encapsulation, the *BI* was improved and amounted to 5.1–8.4% when SA was used, then values of 8.4–12.8% were obtained with SA-GA, and the highest *BI* of TFC were obtained after simulated digestion of SA-GEL microencapsulated powders with values ranging from 8.0 to 14.0% (Figure 8D).

The release of TPA is significantly reduced in grape pomace extracts after OP (0.1–0.8-fold), with a very low release observed in IP (0.10–0.23 mg/100 mg_EXT_) (Figure 9A–C). This indicates that the proanthocyanidins are significantly degraded or absorbed in the earlier stages of digestion. Encapsulation with SA did not significantly alter the trend in TPA release—there was still a marked decrease in the release of TPA across all phases of digestion (Figure 9A–C). For SA-GA microencapsulated powders, the release of TPA in GP is also reduced compared to OP (0.1–0.3-fold) (Figure 9A–C). Furthermore, no change in TPA release in IP compared to GP was observed for CSE-SA-GA microencapsulated powders (Figure 9A), while an increase in TPA release in IP (1.6-fold) was observed for ME-SA-GA powders (Figure 9C). This indicates a certain degree of protective effect of the SA-GA coating combination on TPA. All the SA-GEL microencapsulated powders showed the best retention of TPA during OP and GP and a significant increase in release in IP compared to GP (1.1–3.0-fold) (Figure 9A–C). After simulated digestion of PRE, *BI* of TPA was 1.5–4.0%, and encapsulation with SA increased TPA *BI* to 2.3–5.2%, with the combination of SA-GA to 2.7–5.7% and with SA-GEL to 2.5–7.2% (Figure 9D).

#### 2.5.2. Individual Phenolic Compounds

Table 3 shows the *BI* values only for the individual phenolic compounds from the microencapsulated powders that were detected at the end of the IP digestion phase. The bold values highlight the *BI* values of the individual phenolic compounds that were higher in the microencapsulated powders compared to the *BI* values of non-encapsulated extracts. Otherwise, a cumulative release profile of all quantified individual phenolics in microencapsulated powders and grape pomace extracts can be seen in all three phases of digestion in the Supplementary Materials (Tables S1–S3). The *BI* (Table 3) was calculated from the content of the individual phenolic compound in the tested samples before digestion (Table 1) and at the end of IP (Tables S1–S3) according to Equation (2).

In general, encapsulation had an effect on increasing *BI* values for phenolic acids and flavanols (Table 3) under test conditions compared to non-encapsulated extracts (Tables S1–S3). In addition, the *BI* was higher in most cases when another coating was used in addition to SA. The higher values of *BI* for individual phenolics indicate an effective encapsulation process—protecting the active ingredient in the earlier stages of digestion and allowing significant release upon reaching the intestine where absorption is most desirable.

Of all phenolic acids, encapsulation with tested coatings had the most positive effect on the *BI* values for *o*-coumaric acid, which ranged from 101.4 to 424.2% depending on microencapsulated powders. However, the highest *BI* values were found for the microencapsulated SA-GA powders, which were 2.92–7.8-fold higher than the *BI* values of the non-encapsulated extracts (Tables S1–S3). When the *BI* value exceeds 100%, as is the case for certain phenolic acids, this indicates that more complex phenolic compounds were broken down into simpler compounds during simulated digestion. In the case of hydroxybenzoic acid, encapsulation had the greatest effect on increasing the *BI* values of vanillic and ellagic acid for all microencapsulated samples except the microencapsulated CFE-SA powders. However, the highest *BI* value was achieved with the microencapsulated SA-GEL powders, which ranged from 47.1 to 70.9% for vanillic acid and from 23.6 to 80.7% for ellagic acid, suggesting that SA-GEL may provide better protection and controlled release properties for these acids compared to other encapsulation coatings. For gallic acid, 3,4-dihydroxybenzoic acid and synergic acid, there is no consistent trend of release during digestion and an increase in *BI* in relation to the coating used. The microencapsulated powders CSE-SA-GA and ME-SA-GEL showed the highest increase in *BI* of gallic acid compared to the non-encapsulated extract, with BI values of 32.62% and 88.5%, respectively. For 3,4-dihydroxybenzoic acid, SA-GA and SA-GEL coatings were found to have an effect on increasing *BI*, in contrast to SA alone. The largest increase in *BI* was observed for CFE-SA-GA powders, where the *BI* was 124.5%. The release of syringic acid was minimal in all samples (extracts and microencapsulated powders) or it was not detected, suggesting that it may be more susceptible to degradation and less effectively protected by freeze-drying. An exception is the release of syringic acid from CFE-SA-GA powders where this acid was detected in all gastrointestinal phases and *BI* was determined (78.3%).

Flavanols, especially epicatechin and catechin, play a crucial role due to their health-promoting properties, including cardiovascular benefits and antioxidant activities [57]. When observing the digestion of microencapsulated CSE and ME powders, there was a remarkable increase (3.5–4.9-fold) in epicatechin *BI* compared to grape pomace extracts as well as a higher release rate (Table 3 and Tables S1–S3). This considerable release rate suggests that coatings used effectively delay the release of epicatechin in the upper gastrointestinal tract and allow a sustained release of epicatechin that could be beneficial for ensuring prolonged availability for absorption in the lower gastrointestinal tract. In the case of encapsulated CFE, an improved release profile and stability of epicatechin during digestion was also observed after encapsulation compared to the extract, with the *BI* reaching 14.8–30.6%. Compared to epicatechin, catechin showed less stability and deviation from the desired release profile, so *BI* (20.9%) could only be determined for ME-SA-GEL powder. Beside epicatechin and catechin, other important flavanols such as epicatechin gallate, gallocatechin gallate, procyanidin B1 and procyanidin B2 also have remarkable properties and health benefits and are therefore important to consider in simulated digestion [57]. Epicatechin gallate and gallocatechin gallate showed remarkable stability and release from all microencapsulated powders, achieving *BI* values from 79.1 to 985.9% and 294.6 to 1028.4%, respectively. In general, the highest *BI* values for these compounds were achieved when an SA-GEL coating was used. Procyanidin B1 and B2 was only detectable at very low concentrations or not at all in the later stages of digestion in almost all samples with exception for CFE-SA-GA microencapsulated powders reaching *BI* of 27.6%. The low detection of procyanidins B1 and B2 could indicate their rapid degradation or complexation with other dietary components.

In all samples (Tables S1–S3), stilbenes resveratrol and ε-viniferin, were not detected throughout the simulated digestion in vitro. This indicates that these compounds are degraded during freeze-drying and under digestive conditions, which agrees with the literature that indicates that these compounds can be sensitive to factors such as pH, temperature and enzyme activity that prevail during the digestive process [58].

The quantified flavonols in the grape pomace extracts, quercetin, rutin and kaempferol (Table 1) were minimally released from the extracts and the microencapsulated powders during simulated digestion in vitro, and only rutin was detected in IP after digestion of non-encapsulated CFE, reaching a *BI* of 29.0% (Table S2).

Anthocyanins, a class of water-soluble pigments responsible for the red, purple and blue colors of many fruits and vegetables, have health-promoting properties associated with antioxidant and anti-inflammatory effects [1]. However, their stability and release during digestion can be challenging due to their sensitivity to pH changes and digestive enzymes [59,60]. Encapsulation did not improve the stability during digestion, nor for the *BI* of anthocyanins compared to the non-encapsulated extract (Tables S1–S3).

## 3. Conclusions

The study successfully demonstrates the potential of freeze-drying as a microencapsulation technique using sodium alginate (SA) alone and with additional polymers of natural origin such as gum Arabic (SA-GA) and gelatin (SA-GEL) to improve the bioaccessibility of phenolic compounds from tested grape pomace extracts (CSE; CFE; ME).

The encapsulation efficiency (*EE*, %) was affected by the coating used regardless of the samples tested and was remarkably higher when a combination of polymers was used as a coating compared to SA, and an increase of 8.76–11.33% and 11.17–22.80% was achieved for SA-GA and SA-GEL powders, respectively. The release kinetics of the encapsulated total phenolic compounds was described by the Korsmeyer–Peppas model for all samples, with the exception of the microencapsulated ME-SA-GEL powders, which did not fit any of the mathematical models tested. The physicochemical characterization showed that encapsulation by freeze-drying converted the crystalline structure of the grape pomace extracts into an amorphous form, which in turn had a positive effect on the release and bioaccessibility index (*BI*) of the phenolic compounds in the intestinal phase of simulated digestion. Bioaccessibility was also influenced by the coatings used and, in general, the highest *BI* of phenolic compounds was obtained from SA-GEL microencapsulated powders, highlighting the effectiveness of the encapsulation method in preserving the functional properties of these compounds during the digestion process.

These results suggest that microencapsulation of phenolic-rich grape pomace extracts not only contributes to the upcycling of grape pomace, one of the waste streams of wineries, but also offers a promising avenue for the development of functional foods and dietary supplements that utilize the health benefits of phenolic compounds. Phenolic phytochemicals are potent antioxidants that can mitigate oxidative stress and inflammation and support the health of the elderly or immunocompromised. Furthermore, this study underlines the importance of promoting the circular economy in the food industry. By upcycling grape pomace into valuable extracts, this research also promotes the sustainable use of natural resources. The integration of waste-derived ingredients into food products reflects a move towards more environmentally sustainable and economically viable practices in the industry.

## 4. Materials and Methods

### 4.1. Chemicals and Reagents

For the extraction of phenolic compounds, 96% ethanol (p.a.) was procured from Lab Expert (Shenzhen, Guangdong, China). The coatings used for encapsulation (alginic acid sodium salt (SA) from brown algae (low viscosity), gum Arabic (powder) (GA) and gelatin (GEL) from cold water fish skin) and digestive enzymes pepsin from porcine gastric mucosa (freeze-dried powder, ≥2500 units/mg protein), pancreatin from porcine pancreas (8 × USP) and porcine bile extract, were purchased from Sigma Aldrich (Saint Louis, MO, USA). Salts required for the preparation of electrolyte solutions and buffers were purchased from Acros Organics (Geel, Belgium), Gram Mol (Zagreb, Croatia) and Kemika (Zagreb, Croatia).

Standards used for UHPLC analysis of phenolic compounds, including phenolic acids, flavonols, flavan-3-ols, stilbenes and anthocyanins, were purchased from Sigma Aldrich (Saint Louis, MO, USA), Extrasynthese (Genay, France), Acros Organics (Geel, Belgium), and Applihem (Darmstadt, Germany). UHPLC-grade reagents, including methanol, glacial acetic acid, and acetonitrile, were procured from J.T. Baker (Arnhem, Netherlands), Macron Fine Chemicals (Gliwice, Poland), and Fisher Chemical (Loughborough, United Kingdom). Reagents required for the spectrophotometric determination of total phenolic compounds, total flavonoids and total extractable proanthocyanidins were purchased from CPA chem (Bogomilovo, Bulgaria), Alfa Aesar GmbH & Co KG (Kandel, Germany), and Acros Organics (Geel, Belgium).

### 4.2. Grape Pomace Samples

Grape pomace, the solid residue from wine production, consisting of seeds, pomace and skins, was used. Specifically, the pomace from the Cabernet Sauvignon (CS), Cabernet Franc (CF) and Merlot (M) grape varieties was obtained from the Erdut winery in Erdut, eastern Croatia. After collection, grape pomace samples were air-dried for 48 h at 25–27 °C and stored at room temperature. Before use in the experiments, the pomace was finely ground using an ultracentrifugal mill (Retsch ZM200, Haan, Germany) to achieve a particle size of ≤ 1 mm and the dry matter content was determined as follows: CS—92.91 ± 0.01%, CF—91.95 ± 0.02%, and M—92.15 ± 0.04%.

### 4.3. Preparation of Grape Pomace Phenol-Rich Extracts

Conventional solid–liquid extraction of phenolic compounds from grape pomace samples was performed according to the protocol described by Šelo et al. [61]. In summary, 1 g of grape pomace was subjected to extraction with 40 mL of a 50% aqueous ethanol solution. The extraction process lasted 120 min at 80 °C and 200 rpm in a shaking water bath (Julabo, SW-23, Seelbach, Germany). After extraction, the samples were centrifuged for 10 min at 11,000× *g* (Z 326 K, Hermle Labortechnik GmbH, Germany), and the supernatants were concentrated using a rotary evaporator (Büchi, R-210, Flawil, Switzerland) at 50 °C and 48 mbar to obtain dry grape pomace phenol-rich extracts which were then used for encapsulation, characterization and analysis of in vitro behavior during simulated digestion.

### 4.4. Determination of Total Phenolic Compounds

Total phenolic content (TPC), total flavonoid content (TFC) and total extractable proanthocyanidins (TPA) were determined spectrophotometrically, and phenolic compound content was expressed as mean of three replicates ± standard deviation (SD).

TPC analysis was performed using the Folin–Ciocalteu method modified by Waterhouse [62]. First, 40 µL of the sample was added to 3160 µL of distilled water, followed by 200 µL of the Folin–Ciocalteu reagent. Eight minutes later, 600 µL of sodium carbonate (20% *w*/*v*) was added. The mixture was then incubated at 40 °C for 30 min. The absorbance was measured at 765 nm and compared to a blank prepared with extraction solvent. The results were expressed as gallic acid equivalents per weight of extracts (mg_GAE_/100 mg_EXT_).

TFC was measured using the aluminum chloride method adopted from Marinova et al. [63], but with some modifications. First, 500 µL of the sample was mixed with 2 mL of water, followed by the addition of 150 µL of 5% sodium nitrite (*w*/*v*). After 5 min, 150 µL of 10% aluminum chloride (*w*/*v*) was added and 6 min later, 1 mL of 1 M sodium hydroxide was added. The mixture was then diluted with 1.2 mL distilled water and vortexed. The absorbance was then measured at 510 nm using a water blank instead of the sample. The results were expressed as (+)-catechin equivalents per weight of extracts (mg_CE_/100 mg_EXT_).

TPA was assessed using a modified version of the acid–butanol reaction method described by Škerget et al. [64]. For preparation, a ferrous sulfate heptahydrate solution was mixed in an HCl-butanol solution (2:3, *v*/*v*) and 5 mL of this mixture was added to 500 µL of the sample. After thorough mixing, the samples were incubated in a water bath preheated to 95 °C for 15 min. After incubation, the samples were cooled in water and their absorbance was recorded at 540 nm using a blank prepared with distilled water. The TPA values were calculated based on the molar weight and extinction coefficient of cyanidin and the results were expressed in mg per 100 mg of extracts.

### 4.5. Determination of Individual Phenolic Compounds

Ultra-high performance liquid chromatography (UHPLC Nexera XR, Shimadzu, Japan) was used to quantify various phenolic compounds in grape pomace extracts and extracts containing freeze-dried powders. Compounds such as phenolic acids, flavanols, flavonols, stilbenes and anthocyanins were separated using a reversed-phase Kinetex^®^ C18 core-shell column and detected using a photodiode array (PDA). Prior to analysis, samples were prepared in appropriate solvents and filtered through 0.45 µm membranes. The data were processed using LabSolutions software version 5.87.

The quantification of phenolic acids, flavanols, flavonols and stilbenes was based on the method developed by Bucić-Kojić et al. [65]. A linear gradient of two solvent phases was used: phase A, consisting of 1.0% acetic acid in water, and phase B, consisting of methanol–acetonitrile (50:50, *v*/*v*). Chromatography was performed at a flow rate of 1 mL/min and a temperature of 30 °C. The gradient scheme started with 5% to 30% of phase B over 25 min, increased to 40% over the next 10 min, increased to 48% over 5 min, then to 70% in another 10 min, and finally reached 100% in 5 min. The system was held isocratically at 100% phase B for 5 min, followed by a return to baseline conditions over 10 min and a 12-min period for column equilibration. The injection volume for each sample was set to 20 µL.

According to method described by Bucić-Kojić et al. [66], determination of anthocyanins was performed. Briefly, two mobile phases were used: A. water/formic acid/acetonitrile (87:10:3, *v*/*v*/*v*) and B. water/formic acid/acetonitrile (40:10:50, *v*/*v*/*v*) with a gradient program for 10 min from 10 to 25% in mobile phase B, 5 min from 25 to 31% in mobile phase B, 5 min from 31 to 40% in mobile phase B, 10 min from 40 to 50% in mobile phase B, 10 min from 50 to 100% in mobile phase B, 10 min from 100 to 10% in mobile phase B. The injection volume of the sample was 20 μL with a flow rate 0.8 mL/min.

By comparing UV–Vis spectra and retention times with those of authentic standards analyzed under identical conditions, individual phenolic compounds could be detected and quantified. Calibration curves were generated using these external standards. Anthocyanins were specifically measured at wavelengths of 503, 513, 517, 523, 526 and 531 nm, corresponding to callistephin chloride, kuromanin chloride, peonidin-3-*O*-glucoside chloride, myrtillin chloride, oenin chloride and petunidin chloride, respectively. Hydroxybenzoic acids were determined in the range of 252–280 nm, hydroxycinnamic acids at 276–277 nm, flavanols at 273 to 277 nm, procyanidins at 278 nm, flavonols between 355 and 372 nm and stilbenes at 305 to 323 nm. All tests were performed in triplicate and results were expressed as the average of replicates ± standard deviation (SD).

### 4.6. Encapsulation of Grape Pomace Extracts by Freeze-Drying Method

Grape pomace extracts (CSE, CFE and ME) were prepared for encapsulation by dissolving 1.0 g of each extract in a solution of 30% aqueous ethanol (20.8 mL) and distilled water (79.2 mL) and mixing continuously on a magnetic stirrer for 3 hours. The mixture was then centrifuged at 15,000× *g* for 5 min to remove undissolved extract particles. Subsequently, the clear supernatant (liquid extract) (90 mL) was used for encapsulation to which the different coatings (sodium alginate—SA; combination sodium alginate and gum Arabic—SA-GA; combination sodium alginate and gelatin—SA-GEL) were added. The concentration of SA in liquid extract was 3% (*w*/*v*), while the concentration of GA was 1.6% (*w*/*v*) and od GEL at 5% (*w*/*v*). The mixture of liquid extract and coating was stirred on a magnetic stirrer for 24 hours to ensure complete dissolution of the coating used and to allow sufficient time for the active ingredients and coating to bind. After dissolution of the coating(s), the ethanol contained in the solution mixture of extract and coating(s) was evaporated on a rotary evaporator (Büchi, R-210, Flawil, Switzerland) at 50 °C and 48 mbar for about 15 min. The ethanol-free solution of the extract and coating(s) was then transferred to plastic Petri dishes with lids and the samples were frozen overnight at −80 °C (SWUF Ultra Low Temperature Smart Freezer, Witeg, Wertheim, Germany).

Freeze-drying (Alpha 2-4 LSCplus freeze-dryer, Christ, Osterode am Harz, Germany) was carried out at 0.250 mbar for 24–48 h, depending on the coating(s) used. After completion of the process, the samples were crushed in a mortar with a pestle and the resulting microencapsulated powders were stored in a desiccator.

### 4.7. Determination of Encapsulation Efficiency

To determine the encapsulation efficiency, the content of phenolic compounds in the core (*CPC*) and the content of surface phenolic compounds (*SPC*) of the freeze-dried powders was determined according to Vergara et al. [67] with modifications. For the *CPC*, 50 mg of powder and 1 mL of ethanol/acetic acid/water solution (50:8:42, *v*/*v*/*v*) was vortex-mixed for 1 min and then centrifuged at 14,000× *g* for 2 min. The supernatant was collected and separated for the determination of TPC according to the method described in the section “*Determination of Total Phenolic Compounds*”. Similarly, for the determination of *SPC*, 50 mg of the sample was dispersed in 1 mL of ethanol/methanol (1:1, *v*/*v*) solution. The mixture was vortexed for 1 min, centrifuged at 14,000× *g* for 2 min and the supernatant was separated for the determination of the phenolic compounds content. The results were expressed as milligram gallic acid equivalents per gram of freeze-dried powder (mg_GAE_/g_P_).

The encapsulation efficiency (*EE*, %) was determined using the following Equation (1):

(1) $$EE\;\left(\%\right)=\frac{CPC-SPC}{CPC}\;\cdot 100$$

where *CPC* is the content of total phenolic compounds of freeze-dried microencapsulated powder (mg_GAE_/g_P_) and *SPC* is the surface phenolic compounds content (mg_GAE_/g_P_). The obtained results are presented as the mean values of three replicates ± SD.

### 4.8. Physicochemical Characterization of Grape Pomace Extracts, Coatings and Microencapsulated Powder

#### 4.8.1. Scanning Electron Microscopy (SEM)

To analyze the morphology of the freeze-dried powder, scanning electron microscopy was performed using a Hitachi S4700 instrument (Hitachi Scientific Ltd., Tokyo, Japan). Prior to analysis, the samples were coated with a thin layer of gold-palladium in a sputter coater (Bio-Rad SC 502, VG Microtech, Uckfield, UK). The coated samples were then analyzed by SEM at 10 kV.

#### 4.8.2. X-ray Powder Diffraction (XPRD)

The structure of the grape pomace extracts, the coatings and the freeze-dried powders was analyzed using an X-ray powder diffraction system (BRUKER D8 Advance diffractometer, Karlsruhe, Germany). Cu Kα radiation (λ = 1.5406 Å) was used, and the samples were scanned with a VÅNTEC-1 detector at 40 kV and 40 mA in the 3–40 2*θ* interval. Using DIFFRAC plus EVA software (Version 13.0.0.1, Karlsruhe, Germany), data evaluation including background removal, smoothing and Kα2 stripping was performed.

#### 4.8.3. Differential Scanning Calorimetry (DSC)

A differential scanning calorimeter (Mettler Toledo 821e DSC; Mettler Inc., Schwerzenbach, Switzerland) was used to evaluate the thermal properties of the extracts, coatings and freeze-dried powders. The samples (3–5 mg) were analyzed in a temperature range of 25–300 °C at a heating rate of 10 °C/min and a constant argon flow of 150 mL/min.

### 4.9. Release Studies of Phenolic Compounds

The following protocol was performed according to the INFOGEST protocol [54]: the in vitro enzyme-free release and simulated digestion of extracts (CSE, CFE, ME) and freeze-dried powder, with some modifications described by Martinović et al. [40] and Martinović et al. [68]. The protocol consists of three phases: oral (OP), gastric (GP) and intestinal (IP). Each of these phases consists of an electrolyte solution that mimics the solutions of the human gastrointestinal tract [54].

#### 4.9.1. Enzyme-Free Release Study and Release Kinetics

This study was conducted according to the modified INFOGEST protocol described by Martinović et al. [40]. To summarize, release of phenolic compounds was observed throughout the 243-minute period. The temperature was maintained at 37 °C with constant stirring using a magnetic stirrer. To start OP, 200 mg of freeze-dried powder was mixed with 4 mL of simulated salivary fluid (SSF) and 25 µL of CaCl_2_(H_2_O)_2_. The pH of the mixture was adjusted to 7, and redistilled water was added until the total volume reached 10 mL. After 3 min of OP, 2 mL of the mixture was extracted for TPC analysis and a corresponding volume of SSF was added back to the system. At the end of OP, GP began by adding 8 mL of simulated gastric fluid (SGF) and 5 µL of CaCl_2_(H_2_O)_2_ to the mixture. The pH was then lowered to 3 with 1 M HCl. Further, redistilled water was added to reach a final volume of 20 mL, with this phase extending over 120 min. At specific time intervals (6th, 8th, 23rd, 48th, 63rd, 123rd min), 2 mL of the mixture was removed for analysis and the same volume of SGF was added back. The IP then began with the addition of 16 mL of simulated intestinal fluid (SIF) and 40 µL of CaCl_2_(H_2_O)_2_. The pH was again adjusted to 7, and the total volume was adjusted to 40 mL with distilled water. This phase also lasted 120 min. Sampling and replenishment of SIF was performed during the phase as in the previous OP and GP at specific time intervals (126th, 128th, 143rd, 168th, 183rd, 243rd min).

Mathematical modeling is very effective for analyzing the release of bioactive substances from encapsulated systems. In this context, four mathematical models were used to investigate the release of phenolic components from freeze-dried powders during the enzyme-free in vitro release study. The models used included the first-order model, the Higuchi model, the Hixson–Crowell model and the Korsmeyer–Peppas model. Data analysis was performed using DDSolver [69]. To determine the most accurate model to describe phenol release, three criteria were considered: the adjusted coefficient of determination (R^2^_adj_), the Akaike information criterion (AIC) and the model selection criterion (MSC).

#### 4.9.2. Simulated Digestion In Vitro and Bioaccessibility Index (BI)

Simulated digestion in vitro was performed according to the modified INFOGEST protocol described by Martinović et al. [68] using test tubes, each of which presented a specific time interval of each phase (OP_3_, GP_63_, GP_123_, IP_163_ and IP_243_). These test tubes were placed on a vertical multi-function rotator (PTR-60, Grant-bio Instruments, UK) in a thermostat (TC 135 S, Lovibond, Dortmund, Germany) heated at 37 °C. Approximately 100 mg of extracts or 200 mg of extracts containing freeze-dried powders was added to each tube, followed by the addition of 4 mL of SSF solution and 25 µL of CaCl_2_(H_2_O)_2_ to initiate the oral phase. The pH was adjusted to 7 and the volume was brought to 10 mL with distilled water. After 3 min, the oral phase test tube (OP_3_) was removed and in the remaining tubes, the gastric phase was started by adding 8 mL of SGF solution, 5 µL of CaCl_2_(H_2_O)_2_, 500 µL of pepsin and redistilled water to the volume of 20 mL. The gastric phase was continued, and test tubes were removed from the rotator at certain time intervals (GP_63_, GP_123_). The intestinal phase was then initiated by the addition of 8.5 mL of SIF solution, 40 µL of CaCl_2_(H_2_O)_2_, 5 mL of pancreatin solution, 2.5 mL of bile extract solution and the final mixture volume was adjusted to 40 mL with redistilled water. At IP_163_ and IP_243_, samples were withdrawn from the rotator to end the simulated digestion. Upon removal of the test tube from rotator, it was centrifuged (16,000× *g* at 4 °C for 30 min) and the supernatant was filtered using a 0.45 μm membrane (Syringe filters Spheros Nylon, Agilent Technologies, Santa Clara, CA, USA) and purified by solid phase extraction (SPE) to remove impurities (salts, bile extract, enzyme residues) that can interfere with further analysis. SPE was performed using a modified method according to Kamiloglu et al. [70]. From the centrifuged and filtered sample, 4 mL of filtrate was acidified with 80 µL glacial acetic acid to remove enzyme residues and centrifuged (16,000× *g*, 10 min) to isolate a clear fraction for further purification. Before use, SPE cartridges (Superclean LC-18, 100 mg/1 mL, Sigma Aldrich/Supelco) were conditioned with 6 mL of methanol–glacial acetic acid (1:0.01) and 4 mL of distilled water–glacial acetic acid (1:0.01). The centrifuged samples (3 mL) were then passed through these conditioned cartridges, washed with distilled water (15 mL) and then eluted with methanol (3 mL). In this way, the samples were prepared for the final analysis of the TPC, TFC, TPA and individual phenolic compounds.

The bioaccessibility index (*BI*, %) was calculated using the following Equation (2):

(2) $$BI\;\left(\%\right)=\frac{C_{A}}{C_{B}}\;\cdot 100$$

where *C*_A_ is the content of phenolic compounds in the sample after complete digestion (IP_243_) and *C*_B_ is the content of phenolic compounds in extracts before digestion. The phenolic compound content was expressed per 100 mg of the extract.

### 4.10. Statistical Analysis

In order to determine the statistical significance of the differences between the arithmetic means of the samples, a one-way analysis of variance (ANOVA) was performed using TIBCO Statistica software (TIBCO Software Inc., Version 14.0.0.15, Palo Alto, CA, USA). After determining significant differences, a subsequent post hoc analysis was performed using Duncan’s multiple range test to determine the specific sample groups that showed significant differences (*p* < 0.05) [68]. In the figures, samples from identical populations are identified by uniform alphabetical letters.

## Figures and Tables

**Table 1:** Content of individual phenolic compounds of phenol-rich grape pomace extracts (CSE—Cabernet Sauvignon; CFE—Cabernet Franc; ME—Merlot) determined by UHPLC analysis.

	CSE	CFE	ME
*Phenolic acids* (mg/100 mg_EXT_)			
Gallic acid	161.99 ± 31.68	130.00 ± 10.98	207.79 ± 10.15
3,4-Dihydroxybenzoic acid	62.02 ± 11.60	24.09 ± 1.97	75.63 ± 5.51
*p*-Hydroxybenzoic acid	2.97 ± 0.54	1.00 ± 0.04	2.10 ± 0.09
Syringic acid	88.88 ± 20.39	114.36 ± 3.02	51.89 ± 9.07
Vanillic acid	10.45 ± 0.80	14.83 ± 0.74	12.34 ± 0.25
Ellagic acid	94.72 ± 10.39	81.33 ± 1.23	16.32 ± 0.12
Caffeic acid	1.40 ± 0.04	1.04 ± 0.02	0.75 ± 0.01
Ferulic acid	0.97 ± 0.01	2.52 ± 0.25	0.97 ± 0.08
*o*-Coumaric acid	7.46 ± 1.39	19.48 ± 0.58	9.60 ± 0.60
*p*-Coumaric acid	3.43 ± 0.71	1.44 ± 0.04	3.31 ± 0.20
*Flavanols* (mg/100 mg_EXT_)			
Epicatechin	100.71 ± 11.65	547.27 ± 25.23	248.31 ± 9.17
Catechin	240.87 ± 13.61	527.59 ± 28.62	272.26 ± 0.42
Epicatechin gallate	5.78 ± 2.74	32.46 ± 1.65	8.29 ± 0.04
Gallocatechin gallate	72.53 ± 21.84	127.23 ± 9.54	76.11 ± 4.85
Procyanidin B1	118.52 ± 25.63	317.42 ± 2.59	197.93 ± 16.51
Procyanidin B2	46.57 ± 21.88	126.51 ± 23.32	62.19 ± 2.76
*Flavonols* (mg/100 mg_EXT_)			
Quercetin	214.33 ± 24.44	146.04 ± 3.93	120.95 ± 1.84
Kaempferol	13.22 ± 1.34	10.40 ± 1.00	5.19 ± 0.06
Rutin	13.14 ± 3.60	65.08 ± 5.10	24.13 ± 0.95
*Stilbenes* (mg/100 mg_EXT_)			
Resveratrol	7.91 ± 0.45	8.82 ± 0.40	14.16 ± 0.37
*ε*-Viniferin	22.75 ± 0.58	13.72 ± 0.06	8.53 ± 0.31
*Anthocyanins* (mg/100 mg_EXT_)			
Oenin chloride	91.28 ± 0.72	794.37 ± 21.84	32.83 ± 0.36
Myrtillin chloride	12.09 ± 0.04	35.15 ± 0.00	2.81 ± 0.01
Kuromanin chloride	2.29 ± 0.00	4.88 ± 0.03	1.11 ± 0.15
Petunidin chloride	1.89 ± 0.08	7.77 ± 0.01	0.67 ± 0.03
Callistephin chloride	nd	2.27 ± 0.03	nd
Peonidin-3-*O*-glucoside chloride	9.76 ± 0.02	77.75 ± 7.28	7.17 ± 0.09

nd—compound not identified, EXT—extract. The content of the individual phenolic compounds is given as mean value of replicate (mg/100 mg_EXT_) ± SD.

**Table 2:** Estimated parameters of the applied mathematical models for describing the release kinetics of total phenolic compounds (*k*_1_, *k*_H_, *k*_HC_, *k*_KP_—release constants for the corresponding model; *n*—diffusion exponent) from microencapsulated powders containing extracts of Cabernet Sauvignon, Cabernet Franc and Merlot grape pomace prepared with different coatings, and statistical criteria for model approximation success (R^2^_adj_—adjusted coefficient of determination, AIC—Akaike information criterion, MSC—model selection criterion).

Matematical Models		Release Rate Constants and Statistical Criteria of Model Approximation Success								
		Cabernet Sauvignon			Cabernet Franc			Merlot		
		SA	SA-GA	SA-GEL	SA	SA-GA	SA-GEL	SA	SA-GA	SA-GEL
**First Order** **Model**	R^2^_adj_	−0.464	−0.073	−0.641	−0.558	0.021	−0.178	−0.364	−0.079	−1.479
	AIC	134.382	131.764	138.136	136.299	130.475	133.887	134.748	130.362	147.222
	MSC	−2.332	−1.580	−1.568	−2.248	−1.492	−0.784	−1.684	−1.656	−2.129
	*k*_1_	0.040	0.048	0.031	0.041	0.049	0.013	0.026	0.043	0.024
**Higuchi** **Model**	R^2^_adj_	−1.004	−0.501	−0.538	−1.118	−0.477	0.121	−0.498	−0.463	−1.226
	AIC	138.896	136.550	137.223	140.826	136.275	129.785	136.079	134.624	145.714
	MSC	−2.655	−1.922	−1.503	−2.571	−1.906	−0.491	−1.779	−1.961	−2.021
	*k*_H_	8.639	8.851	7.996	8.811	8.839	6.725	8.473	8.474	9.054
**Hixson–** **Crowell Model**	R^2^_adj_	−1.273	−0.790	−0.913	−1.380	−0.826	−0.224	−0.635	−0.800	−1.668
	AIC	140.664	139.010	140.282	142.449	139.223	134.428	137.308	137.528	148.251
	MSC	−2.781	−2.098	−1.722	−2.687	−2.116	−0.823	−1.867	−2.168	−2.202
	*k*_HC_	0.007	0.007	0.006	0.007	0.007	0.003	0.006	0.007	0.006
Korsmeyer–Peppas Model	R^2^_adj_	**0.963**	**0.873**	**0.611**	**0.910**	**0.922**	**0.462**	**0.889**	**0.951**	-
	AIC	**83.525**	**102.775**	**118.761**	**97.381**	**95.742**	**123.825**	**100.523**	**87.613**	-
	MSC	**1.300**	**0.490**	**−0.195**	**0.532**	**0.989**	**−0.065**	**0.761**	**1.397**	-
	*k*_KP_	73.698	72.207	75.257	77.047	67.023	48.962	61.468	64.742	-
	*n*	0.056	0.050	0.014	0.049	0.078	0.093	0.084	0.077	-

SA—sodium alginate; SA-GA—combinations of sodium alginate with gum Arabic; SA-GEL—combinations of sodium alginate and gelatin; ˝-˝—model parameters could not be determined. The statistical criteria of the model that best describe the release of total phenolic compounds from freeze-dried microencapsulated powders with different coatings are marked in bold.

**Table 3:** Bioaccessibility index (*BI*, %) of individual phenolic compounds of microencapsulated extract of Cabernet Sauvignon grape pomace (CSE), Cabernet Franc grape pomace (CFE) and Merlot (ME) grape pomace.

Component		Sample *	Bioaccessibility Index, *BI* (%)		
			CSE	CFE	ME
*Phenolic acids*	Gallic acid	SA	4.8 ± 0.6	12.3 ± 0.6	14.5 ± 0.1
		SA-GA	**32.6 ± 0.5**	69.8 ± 0.9	46.2 ± 0.0
		SA-GEL	**26.2 ± 4.4**	7.3 ± 0.0	**88.5 ± 0.3**
	3,4-Dihydroxybenzoic acid	SA	10.3 ± 0.3	35.7 ± 0.2	11.4 ± 0.1
		SA-GA	**28.8 ± 0.7**	**124.5 ± 2.1**	**31.1 ± 0.0**
		SA-GEL	**31.5 ± 0.2**	66.0 ± 0.0	**47.5 ± 0.7**
	Syringic acid	SA	0.0	0.0	0.0
		SA-GA	0.0	**78.3 ± 8.5**	0.0
		SA-GEL	0.0	0.0	0.0
	Vanillic acid	SA	**42.3 ± 5.9**	26.2 ± 0.9	**32.7 ± 2.4**
		SA-GA	**57.4 ± 1.9**	**35.2 ± 0.4**	**52.9 ± 0.0**
		SA-GEL	**70.9 ± 5.9**	**47.1 ± 0.0**	**53.1 ± 0.8**
	Ellagic acid	SA	**13.4 ± 0.4**	**16.5 ± 0.2**	**19.2 ± 0.3**
		SA-GA	**6.5 ± 0.1**	**11.9 ± 0.6**	**37.5 ± 1.2**
		SA-GEL	**27.3 ± 0.7**	**23.6 ± 0.0**	**80.7 ± 3.1**
	*o*-Coumaric acid	SA	**209.8 ± 4.1**	**110.6 ± 3.4**	**161.4 ± 0.9**
		SA-GA	**353.2 ± 5.2**	**424.2 ± 13.2**	**242.2 ± 0.0**
		SA-GEL	**207.2 ± 23.4**	**101.4 ± 0.0**	**247.4 ± 25.4**
*Flavanols*	Epicatechin	SA	**259.4 ± 6.4**	**30.6 ± 0.2**	**89.0 ± 1.6**
		SA-GA	**245.6 ± 8.7**	**14.8 ± 0.4**	**91.7 ± 0.0**
		SA-GEL	**222.6 ± 0.1**	**30.1 ± 0.3**	**90.1 ± 6.5**
	Catechin	SA	0.0	0.0	0.0
		SA-GA	0.0	0.0	0.0
		SA-GEL	0.0	0.0	**20.9 ± 0.4**
	Epicatechin gallate	SA	**582.6 ± 49.5**	**79.1 ± 3.3**	**332.4 ± 13.0**
		SA-GA	**659.9 ± 3.4**	**159.7 ± 8.7**	**474.6 ± 0.0**
		SA-GEL	**958.9 ± 12.4**	**168.9 ± 0.0**	**713.7 ± 29.5**
	Gallocatechin gallate	SA	**456.2 ± 11.8**	**294.6 ± 4.6**	**365.2 ± 3.4**
		SA-GA	**660.3 ± 17.6**	**353.8 ± 1.1**	**581.2 ± 0.0**
		SA-GEL	**987.3 ± 7.9**	**473.0 ± 0.0**	**1028.4 ± 10.0**
	Procyanidin B2	SA	0.0	0.0	0.0
		SA-GA	0.0	**27.6 ± 5.0**	0.0
		SA-GEL	0.0	0.0	0.0

* (SA)—sodium alginate microencapsulated powder, (SA-GA)—sodium alginate with gum Arabic microencapsulated powder, (SA-GEL)—sodium alginate with gelatin microencapsulated powder. The *BI* values in bold for microencapsulated powder samples indicate higher *BI* values compared to non-encapsulated grape pomace extract of the same origin.

## Data Availability

Data are contained within the article and Supplementary materials.

## Supplementary Materials

The following supporting information can be downloaded at: https://www.mdpi.com/article/10.3390/gels10060353/s1, Figure S1: Cumulative release of total phenolic compounds (TPC) from freeze-dried microencapsulated powders containing grape pomace phenol-rich extracts (A.—Cabernet Sauvignon, B.—Cabernet Franc and C.—Merlot) coated with sodium alginate (SA), sodium alginate with gum arabica (SA-GA) and sodium alginate with gelatin (SA-GEL) expressed as mg gallic acid equivalent (GAE) per g of freeze-dried microencapsulated powders (mg_GAE_/g_P_); Figure S2: Kinetics of phenolic compound release from the freeze-dried microencapsulated powders containing grape pomace extracts of Cabernet Sauvignon, Cabernet Franc and Merlot varieties prepared with different coatings (NA—sodium alginate; SA-GA—combination of sodium alginate and gum Arabic; SA-GEL—combination of sodium alginate ang gelatin; symbols—experimental data, lines—approximate curves according to different mathematical models); Table S1: Content of individual phenolic compounds of phenol-rich grape pomace extract Cabernet Sauvignon (CSE), sodium alginate microencapsulated powder (SA), sodium alginate with gum Arabic microencapsulated powder (SA-GA), and sodium alginate with gelatin microencapsulated powder (SA-GEL) during three phases of in vitro simulated digestion; Table S2: Content of individual phenolic compounds of phenol-rich grape pomace extract Cabernet Franc (CFE), sodium alginate microencapsulated powder (SA), sodium alginate with gum Arabic microencapsulated powder (SA-GA), and sodium alginate with gelatin microencapsulated powder (SA-GEL) during three phases of in vitro simulated digestion; Table S3: Content of individual phenolic compounds of phenol-rich grape pomace extract Merlot (ME), sodium alginate microencapsulated powder (SA), sodium alginate with gum Arabic microencapsulated powder (SA-GA), and sodium alginate with gelatin microencapsulated powder (SA-GEL) during three phases of in vitro simulated digestion.

## References

[1] Sirohi R., Tarafdar A., Singh S., Negi T., Gaur V.K., Gnansounou E., Bharathiraja B. (2020). Green Processing and Biotechnological Potential of Grape Pomace: Current Trends and Opportunities for Sustainable Biorefinery. Bioresour. Technol..

[2] Beres C., Costa G.N.S., Cabezudo I., Da Silva-James N.K., Teles A.S.C., Cruz A.P.G., Mellinger-Silva C., Tonon R.V., Cabral L.M.C., Freitas S.P. (2017). Towards Integral Utilization of Grape Pomace from Winemaking Process: A Review. Waste Manag..

[3] Haminiuk C.W.I., Maciel G.M., Plata-Oviedo M.S.V., Peralta R.M. (2012). Phenolic Compounds in Fruits—An Overview. Int. J. Food Sci. Technol..

[4] Grgić J., Šelo G., Planinić M., Tišma M., Bucić-Kojić A. (2020). Role of the Encapsulation in Bioavailability of Phenolic Compounds. Antioxidants.

[5] Munin A., Edwards-Lévy F. (2011). Encapsulation of Natural Polyphenolic Compounds; a Review. Pharmaceutics.

[6] Xu Y., Yan X., Zheng H., Li J., Wu X., Xu J., Zhen Z., Du C. (2024). The Application of Encapsulation Technology in the Food Industry: Classifications, Recent Advances, and Perspectives. Food Chem..

[7] Bruschi M.L. (2015). Strategies to Modify the Drug Release from Pharmaceutical Systems.

[8] Peppas N.A., Narasimhan B. (2014). Mathematical Models in Drug Delivery: How Modeling Has Shaped the Way We Design New Drug Delivery Systems. J. Control. Release.

[9] Rockenbach I.I., Rodrigues E., Gonzaga L.V., Caliari V., Genovese M.I., Gonçalves A.E.D.S.S., Fett R. (2011). Phenolic Compounds Content and Antioxidant Activity in Pomace from Selected Red Grapes (*Vitis vinifera* L. and *Vitis labrusca* L.) Widely Produced in Brazil. Food Chem..

[10] Iora S.R.F., Maciel G.M., Zielinski A.A.F., Da Silva M.V., Pontes P.V.D.A., Haminiuk C.W.I., Granato D. (2015). Evaluation of the Bioactive Compounds and the Antioxidant Capacity of Grape Pomace. Int. J. Food Sci. Technol..

[11] Xu Y., Burton S., Kim C., Sismour E. (2016). Phenolic Compounds, Antioxidant, and Antibacterial Properties of Pomace Extracts from Four Virginia-grown Grape Varieties. Food Sci. Nutr..

[12] Jin Q., O’Hair J., Stewart A.C., O’Keefe S.F., Neilson A.P., Kim Y.-T., McGuire M., Lee A., Wilder G., Huang H. (2019). Compositional Characterization of Different Industrial White and Red Grape Pomaces in Virginia and the Potential Valorization of the Major Components. Foods.

[13] Yang K., Zhang L., Liao P., Xiao Z., Zhang F., Sindaye D., Xin Z., Tan C., Deng J., Yin Y. (2020). Impact of Gallic Acid on Gut Health: Focus on the Gut Microbiome, Immune Response, and Mechanisms of Action. Front. Immunol..

[14] Ramachandran V., Raja B. (2010). Protective Effects of Syringic Acid against Acetaminophen-Induced Hepatic Damage in Albino Rats. J. Basic Clin. Physiol. Pharmacol..

[15] Lee M.-H., Kang H., Lee K., Yang G., Ham I., Bu Y., Kim H., Choi H.-Y. (2013). The Aerial Part of Taraxacum Coreanum Extract Has an Anti-Inflammatory Effect on Peritoneal Macrophages In Vitro and Increases Survival in a Mouse Model of Septic Shock. J. Ethnopharmacol..

[16] Wei X., Chen D., Yi Y., Qi H., Gao X., Fang H., Gu Q., Wang L., Gu L. (2012). Syringic Acid Extracted from *Herba dendrobii* Prevents Diabetic Cataract Pathogenesis by Inhibiting Aldose Reductase Activity. J. Evid. Based Complementary Altern. Med..

[17] Güven M., Aras A.B., Topaloğlu N., Özkan A., Şen H.M., Kalkan Y., Okuyucu A., Akbal A., Gökmen F., Coşar M. (2015). The Protective Effect of Syringic Acid on Ischemia Injury in Rat Brain. Turk. J. Med. Sci..

[18] Liu Y., Sun C., Li W., Adu-Frimpong M., Wang Q., Yu J., Xu X. (2019). Preparation and Characterization of Syringic Acid–Loaded TPGS Liposome with Enhanced Oral Bioavailability and In Vivo Antioxidant Efficiency. AAPS PharmSciTech.

[19] Alfei S., Marengo B., Zuccari G. (2020). Oxidative Stress, Antioxidant Capabilities, and Bioavailability: Ellagic Acid or Urolithins?. Antioxidants.

[20] Song J., He Y., Luo C., Feng B., Ran F., Xu H., Ci Z., Xu R., Han L., Zhang D. (2020). New Progress in the Pharmacology of Protocatechuic Acid: A Compound Ingested in Daily Foods and Herbs Frequently and Heavily. Pharmacol. Res..

[21] Kowczyk-Sadowy M., Świsłocka R., Lewandowska H., Piekut J., Lewandowski W. (2015). Spectroscopic (FT-IR, FT-Raman, 1H- and 13C-NMR), Theoretical and Microbiological Study of Trans *o*-Coumaric Acid and Alkali Metal *o*-Coumarates. Molecules.

[22] Kiliç I., Yeşiloğlu Y. (2013). Spectroscopic Studies on the Antioxidant Activity of P-Coumaric Acid. Spectrochim. Acta A Mol. Biomol. Spectrosc..

[23] Oniszczuk A. (2016). LC-ESI-MS/MS Analysis and Extraction Method of Phenolic Acids from Gluten-Free Precooked Buckwheat Pasta. Food Anal. Methods.

[24] Anderson J.W. (2004). Whole Grains and Coronary Heart Disease: The Whole Kernel of Truth. Am. J. Clin. Nutr..

[25] Morris M.C. (2002). Dietary Intake of Antioxidant Nutrients and the Risk of Incident Alzheimer Disease in a Biracial Community Study. J. Am. Med. Assoc..

[26] Georgiev V., Ananga A., Tsolova V. (2014). Recent Advances and Uses of Grape Flavonoids as Nutraceuticals. Nutrients.

[27] Heptinstall S., May J., Fox S., Kwik-Uribe C., Zhao L. (2006). Cocoa Flavanols and Platelet and Leukocyte Function: Recent In Vitro and Ex Vivo Studies in Healthy Adults. J. Cardiovasc. Pharmacol..

[28] Yunhai L., Jianguo F., Ting L., Wenqing W., Aihua L. (2003). Anti-Endotoxic Effects of Syringic Acid of Radix Isatidis. Curr. Med. Sci..

[29] Xu Z., Du P., Meiser P., Jacob C. (2012). Proanthocyanidins: Oligomeric Structures with Unique Biochemical Properties and Great Therapeutic Promise. Nat. Prod. Commun..

[30] Beecher G.R. (2004). Proanthocyanidins: Biological Activities Associated with Human Health. Pharm. Biol..

[31] Nandakumar V., Singh T., Katiyar S.K. (2008). Multi-Targeted Prevention and Therapy of Cancer by Proanthocyanidins. Cancer Lett..

[32] Shen D., Feng Y., Zhang X., Gong L., Liu J., Li Y., Liao H. (2022). Antiosteoporosis Studies of 20 Medicine Food Homology Plants Containing Quercetin, Rutin, and Kaempferol: TCM Characteristics, In Vivo and In Vitro Activities, Potential Mechanisms, and Food Functions. J. Evid. Based Complement. Altern. Med..

[33] Guerrero R.F., Biais B., Richard T., Puertas B., Waffo-Teguo P., Merillon J.-M., Cantos-Villar E. (2016). Grapevine Cane’s Waste Is a Source of Bioactive Stilbenes. Ind. Crops Prod..

[34] Castellanos-Gallo L., Ballinas-Casarrubias L., Espinoza-Hicks J.C., Hernández-Ochoa L.R., Muñoz-Castellanos L.N., Zermeño-Ortega M.R., Borrego-Loya A., Salas E. (2022). Grape Pomace Valorization by Extraction of Phenolic Polymeric Pigments: A Review. Processes.

[35] Flamini R., Mattivi F., Rosso M., Arapitsas P., Bavaresco L. (2013). Advanced Knowledge of Three Important Classes of Grape Phenolics: Anthocyanins, Stilbenes and Flavonols. Int. J. Mol. Sci..

[36] do Socorro S., Chagas M., Behrens M.D., Moragas-Tellis C.J., Penedo G.X.M., Silva A.R., Gonçalves-de-Albuquerque C.F. (2022). Flavonols and Flavones as Potential Anti-Inflammatory, Antioxidant, and Antibacterial Compounds. Oxid. Med. Cell. Longev..

[37] Luo Q. (2022). Gelatin-Based Composite Films and Their Application in Food Packaging: A Review. J. Food Eng..

[38] Li Y., Lim L.-T., Kakuda Y. (2009). Electrospun Zein Fibers as Carriers to Stabilize (−)-Epigallocatechin Gallate. J. Food Sci..

[39] Jyothi N.V.N., Prasanna P.M., Sakarkar S.N., Prabha K.S., Ramaiah P.S., Srawan G.Y. (2010). Microencapsulation Techniques, Factors Influencing Encapsulation Efficiency. J. Microencapsul..

[40] Martinović J., Lukinac J., Jukić M., Ambrus R., Planinić M., Šelo G., Perković G., Bucić-Kojić A. (2023). The Release of Grape Pomace Phenolics from Alginate-Based Microbeads during Simulated Digestion In Vitro: The Influence of Coatings and Drying Method. Gels.

[41] Mariod A.A. (2018). Functional Properties of Gum Arabic. Gum Arabic.

[42] Tyuftin A.A., Kerry J.P. (2021). Gelatin Films: Study Review of Barrier Properties and Implications for Future Studies Employing Biopolymer Films. Food Packag. Shelf Life.

[43] Ballesteros L.F., Ramirez M.J., Orrego C.E., Teixeira J.A., Mussatto S.I. (2017). Encapsulation of Antioxidant Phenolic Compounds Extracted from Spent Coffee Grounds by Freeze-Drying and Spray-Drying Using Different Coating Materials. Food Chem..

[44] Ravichai K., Muangrat R. (2019). Effect of Different Coating Materials on Freeze-drying Encapsulation of Bioactive Compounds from Fermented Tea Leaf Wastewater. J. Food Process. Preserv..

[45] Zuidam N.J., Shimoni E., Zuidam N.J., Nedovic V. (2010). Chapter 2: Overview of Microencapsulates for Use in Food Products or Processes and Methods to Make Them. Encapsulation Technologies for Active Food Ingredients and Food Processing.

[46] Papoutsis K., Golding J., Vuong Q., Pristijono P., Stathopoulos C., Scarlett C., Bowyer M. (2018). Encapsulation of Citrus By-Product Extracts by Spray-Drying and Freeze-Drying Using Combinations of Maltodextrin with Soybean Protein and ι-Carrageenan. Foods.

[47] Tao Y., Wang P., Wang J., Wu Y., Han Y., Zhou J. (2017). Combining Various Wall Materials for Encapsulation of Blueberry Anthocyanin Extracts: Optimization by Artificial Neural Network and Genetic Algorithm and a Comprehensive Analysis of Anthocyanin Powder Properties. Powder Technol..

[48] Arab K., Ghanbarzadeh B., Ayaseh A., Jahanbin K. (2021). Extraction, Purification, Physicochemical Properties and Antioxidant Activity of a New Polysaccharide from *Ocimum album* L. Seed. Int. J. Biol. Macromol..

[49] Archana G., Sabina K., Babuskin S., Radhakrishnan K., Fayidh M.A., Babu P.A.S., Sivarajan M., Sukumar M. (2013). Preparation and Characterization of Mucilage Polysaccharide for Biomedical Applications. Carbohydr. Polym..

[50] Devi N., Kakati D.K. (2013). Smart Porous Microparticles Based on Gelatin/Sodium Alginate Polyelectrolyte Complex. J. Food Eng..

[51] Boostani S., Jafari S.M. (2021). A Comprehensive Review on the Controlled Release of Encapsulated Food Ingredients; Fundamental Concepts to Design and Applications. Trends Food Sci. Technol..

[52] Luchese C.L., Uranga J., Spada J.C., Tessaro I.C., De La Caba K. (2018). Valorisation of Blueberry Waste and Use of Compression to Manufacture Sustainable Starch Films with Enhanced Properties. Int. J. Biol. Macromol..

[53] Shahidi F., Dissanayaka C.S. (2023). Phenolic-Protein Interactions: Insight from in-Silico Analyses—A Review. Food Prod. Process. Nutr..

[54] Brodkorb A., Egger L., Alminger M., Alvito P., Assunção R., Ballance S., Bohn T., Bourlieu-Lacanal C., Boutrou R., Carrière F. (2019). INFOGEST Static In Vitro Simulation of Gastrointestinal Food Digestion. Nat. Protoc..

[55] Carbonell-Capella J.M., Buniowska M., Barba F.J., Esteve M.J., Frígola A. (2014). Analytical Methods for Determining Bioavailability and Bioaccessibility of Bioactive Compounds from Fruits and Vegetables: A Review. Comp. Rev. Food Sci. Food Safe.

[56] Quirós-Sauceda A.E., Palafox-Carlos H., Sáyago-Ayerdi S.G., Ayala-Zavala J.F., Bello-Perez L.A., Álvarez-Parrilla E., De La Rosa L.A., González-Córdova A.F., González-Aguilar G.A. (2014). Dietary Fiber and Phenolic Compounds as Functional Ingredients: Interaction and Possible Effect after Ingestion. Food Funct..

[57] González-Manzano S., Rivas-Gonzalo J.C., Santos-Buelga C. (2004). Extraction of Flavan-3-Ols from Grape Seed and Skin into Wine Using Simulated Maceration. Anal. Chim. Acta.

[58] Fuloria S., Sekar M., Khattulanuar F.S., Gan S.H., Rani N.N.I.M., Ravi S., Subramaniyan V., Jeyabalan S., Begum M.Y., Chidambaram K. (2022). Chemistry, Biosynthesis and Pharmacology of Viniferin: Potential Resveratrol-Derived Molecules for New Drug Discovery, Development and Therapy. Molecules.

[59] Kamonpatana K., Giusti M.M., Chitchumroonchokchai C., MorenoCruz M., Riedl K.M., Kumar P., Failla M.L. (2012). Susceptibility of Anthocyanins to Ex Vivo Degradation in Human Saliva. Food Chem..

[60] He J., Giusti M.M. (2010). Anthocyanins: Natural Colorants with Health-Promoting Properties. Annu. Rev. Food Sci. Technol..

[61] Šelo G., Planinić M., Tišma M., Martinović J., Perković G., Bucić-Kojić A. (2023). Bioconversion of Grape Pomace with Rhizopus Oryzae under Solid-State Conditions: Changes in the Chemical Composition and Profile of Phenolic Compounds. Microorganisms.

[62] Waterhouse A.L. (2001). Determination of Total Phenolics. Current Protocols in Food Analytical Chemistry.

[63] Marinova D., Ribarova F., Atanassova M. (2005). Total Phenolics And Total Flavonoids In Bulgarian Fruits And Vegetables. J. Univ. Chem. Technol. Metall..

[64] Škerget M., Kotnik P., Hadolin M., Hraš A.R., Simonič M., Knez Ž. (2005). Phenols, Proanthocyanidins, Flavones and Flavonols in Some Plant Materials and Their Antioxidant Activities. Food Chem..

[65] Bucić-Kojić A., Šelo G., Zelić B., Planinić M., Tišma M. (2017). Recovery of Phenolic Acid and Enzyme Production from Corn Silage Biologically Treated by *Trametes versicolor*. Appl. Biochem. Biotechnol..

[66] Bucić-Kojić A., Fernandes F., Silva T., Planinić M., Šelo G., Šibalić D., Pereira D.M., Andrade P.B. (2020). Enhancement of the Anti-Inflammatory Properties of Grape Pomace Treated by *Trametes versicolor*. Food. Funct..

[67] Vergara C., Saavedra J., Sáenz C., García P., Robert P. (2014). Microencapsulation of Pulp and Ultrafiltered Cactus Pear (Opuntia Ficus-Indica) Extracts and Betanin Stability during Storage. Food Chem..

[68] Martinović J., Lukinac J., Jukić M., Ambrus R., Planinić M., Šelo G., Klarić A.-M., Perković G., Bucić-Kojić A. (2023). In Vitro Bioaccessibility Assessment of Phenolic Compounds from Encapsulated Grape Pomace Extract by Ionic Gelation. Molecules.

[69] Zhang Y., Huo M., Zhou J., Zou A., Li W., Yao C., Xie S. (2010). DDSolver: An Add-In Program for Modeling and Comparison of Drug Dissolution Profiles. AAPS J. PharmSciTech.

[70] Kamiloglu S., Ozkan G., Isik H., Horoz O., Van Camp J., Capanoglu E. (2017). Black Carrot Pomace as a Source of Polyphenols for Enhancing the Nutritional Value of Cake: An In Vitro Digestion Study with a Standardized Static Model. LWT—Food Sci. Technol..

