# Supplementary material for: Microencapsulation of Grape Pomace Extracts with Alginate-Based Coatings by Freeze-Drying: Release Kinetics and In Vitro Bioaccessibility Assessment of Phenolic Compounds
Source: Gels. 2024 May 21;10(6):353. doi: 10.3390/gels10060353 (PMC11203361; doi:10.3390/gels10060353)
Supplement: Supplementary file 1 [file gels-10-00353-s001.zip › gels-3017868-supplementary.pdf]

Supplementary Materials

Microencapsulation of Grape Pomace Extracts with Alginate-Based Coatings by Freeze-Drying: Release Kinetics and In Vitro Bioaccessibility Assessment of Phenolic Compounds

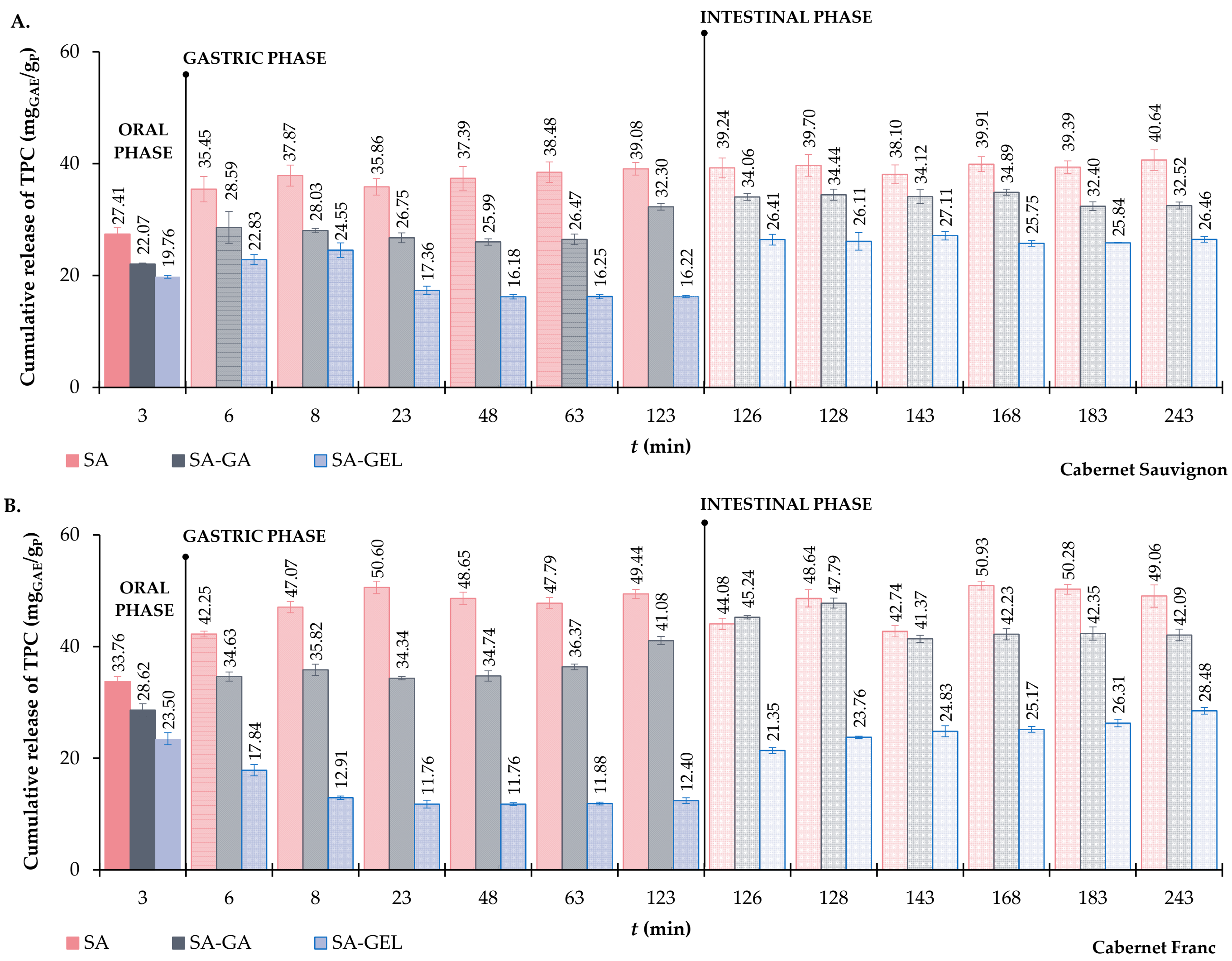
A.
Cumulative release of TPC (mgGAE/gP)
ORAL PHASE
GASTRIC PHASE
INTESTINAL PHASE
27.41 22.07 19.76
35.45 28.59 22.83
37.87 28.03 24.55
35.86 26.75 17.36
37.39 25.99 16.18
38.48 26.47 16.25
39.08 32.30 16.22
39.24 34.06 26.41
39.70 34.44 26.11
38.10 34.12 27.11
39.91 34.89 25.75
39.39 32.40 25.84
40.64 32.52 26.46
3 6 8 23 48 63 123 126 128 143 168 183 243
t (min)
SA SA-GA SA-GEL
Cabernet Sauvignon
B.
Cumulative release of TPC (mgGAE/gP)
ORAL PHASE
GASTRIC PHASE
INTESTINAL PHASE
33.76 28.62 23.50
42.25 34.63 17.84
47.07 35.82 12.91
50.60 34.34 11.76
48.65 34.74 11.76
47.79 36.37 11.88
49.44 41.08 12.40
44.08 45.24 21.35
48.64 47.79 23.76
42.74 41.37 24.83
50.93 42.23 25.17
50.28 42.35 26.31
49.06 42.09 28.48
3 6 8 23 48 63 123 126 128 143 168 183 243
t (min)
SA SA-GA SA-GEL
Cabernet Franc

C.

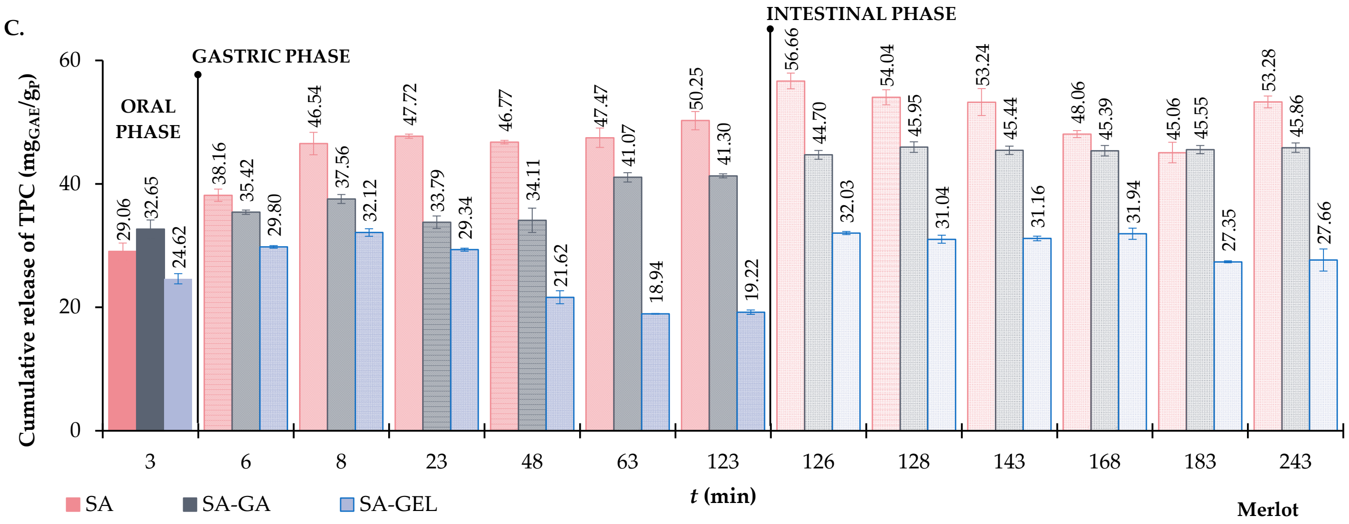

Figure S1. Cumulative release of total phenolic compounds (TPC) from freeze-dried microencapsulated powders containing grape pomace phenol-rich extracts (A. – Cabernet Sauvignon, B. – Cabernet Franc and C. – Merlot) coated with sodium alginate (SA), sodium alginate with gum arabica (SA-GA) and sodium alginate with gelatin (SA-GEL) expressed as mg gallic acid equivalent (GAE) per g of freeze-dried microencapsulated powders (mg_{GAE}/g_P).

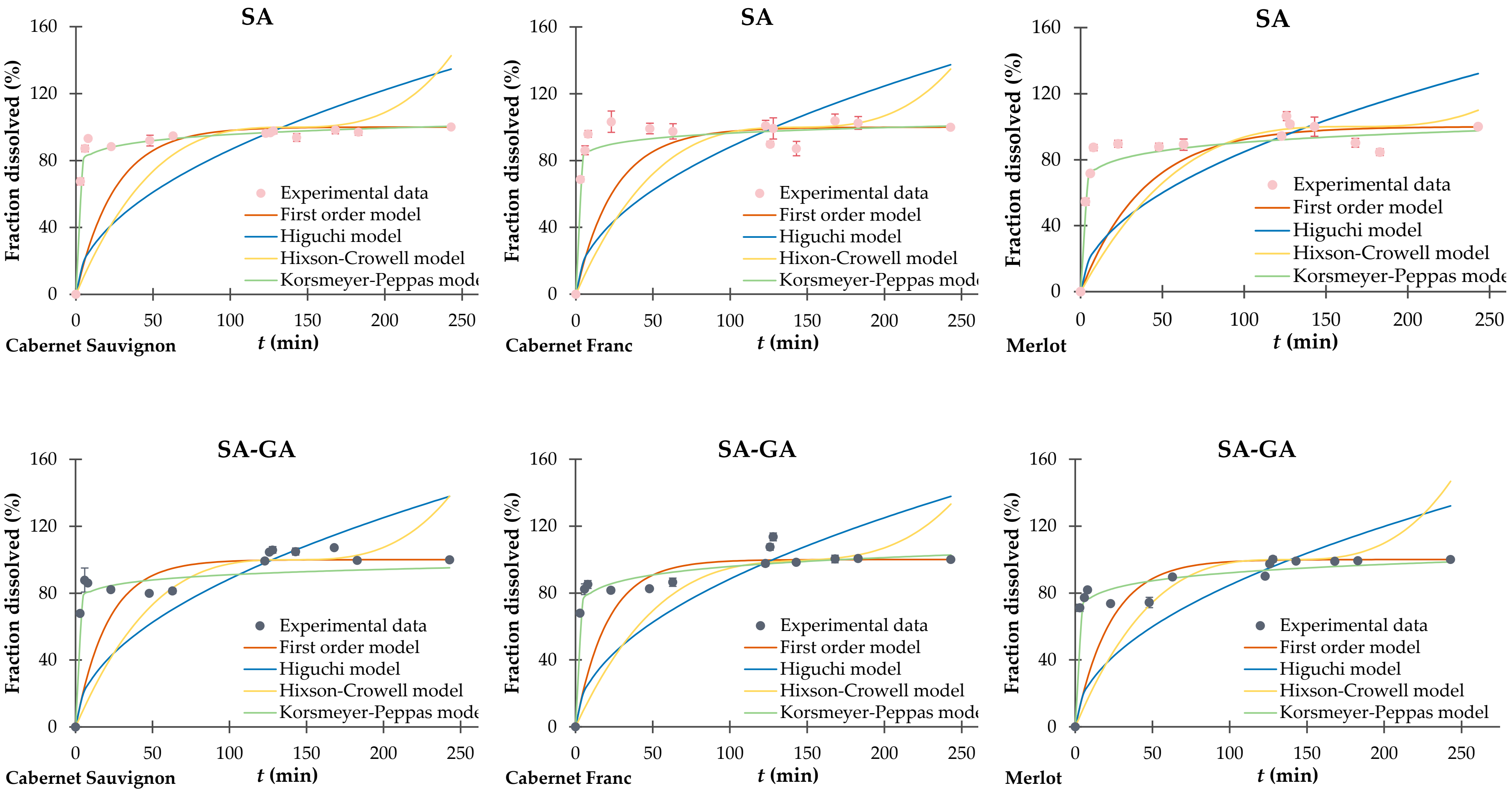

SA
160
120
80
40
0
Fraction dissolved (%)
0
50
100
150
200
250
t (min)
Cabernet Sauvignon
Experimental data
First order model
Higuchi model
Hixson-Crowell model
Korsmeyer-Peppas mode
SA
Fraction dissolved (%)
t (min)
Cabernet Franc
Experimental data
First order model
Higuchi model
Hixon-Crowell model
Korsmeyer-Peppas mod
SA
Fraction dissolved (%)
t (min)
Merlot
Experimental data
First order model
Higuchi model
Hixson-Crowell model
Korsmeyer-Peppas mode
SA-GA
Fraction dissolved (%)
t (min)
Cabernet Sauvignon
Experimental data
First order model
Higuchi model
Hixson-Crowell model
Korsmeyer-Peppas mode
SA-GA
Fraction dissolved (%)
t (min)
Cabernet Franc
Experimental data
First order model
Higuchi model
Hixson-Crowell model
Korsmeyer-Peppas mod
SA-GA
Fraction dissolved (%)
t (min)
Merlot
Experimental data
First order model
Higuchi model
Hixson-Crowell model
Korsmeyer-Peppas model

Continuing **Figure S2.**

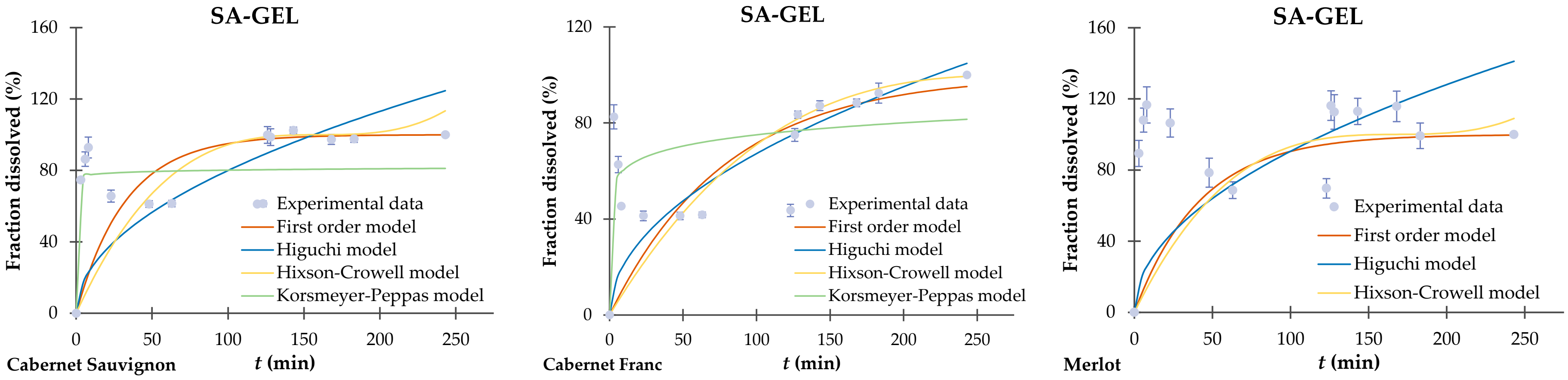

Figure S2. Kinetics of phenolic compound release from the freeze-dried microencapsulated powders containing grape pomace extracts of Cabernet Sauvignon, Cabernet Franc and Merlot varieties prepared with different coatings (NA – sodium alginate; SA-GA – combination of sodium alginate and gum Arabic; SA-GEL – combination of sodium alginate ang gelatin; symbols – experimental data, lines – approximate curves according to different mathematical models).

Table S1. Content of individual phenolic compounds of phenol-rich grape pomace extract Cabernet Sauvignon (CSE), sodium alginate microencapsulated powder (SA), sodium alginate with gum Arabic microencapsulated powder (SA-GA), and sodium alginate with gelatin microencapsulated powder (SA-GEL) during three phases of in vitro simulated digestion.

**Component**	**Sample**	**Oral phase**	**Gastric phase**		**Intestinal phase**		**Bioaccessibility index**
		**OP_3**	**GP_{63}**	**GP_{123}**	**IP_{183}**	**IP_{243}**	***BI* (%)**
***Phenolic acids* (μg/100 mg_{EXT})**							
Gallic acid	CSE	0.70 ± 0.04	-	1.25 ± 0.01	-	36.26 ± 1.29	22.4 ± 0.8
	SA	3.49 ± 0.37	14.19 ± 0.70	9.94 ± 0.11	37.44 ± 1.89	7.81 ± 0.92	4.8 ± 0.6
	SA-GA	1.81 ± 0.02	7.77 ± 0.00	13.98 ± 1.69	66.39 ± 0.39	52.84 ± 0.84	32.6 ± 0.5
	SA-GEL	3.66 ± 0.03	6.93 ± 0.10	5.85 ± 0.36	41.98 ± 1.13	42.46 ± 7.08	26.2 ± 4.4
3,4-Dihydroxybenzoic acid	CSE	nd	-	nd	-	11.36 ± 0.37	18.3 ± 0.6
	SA	nd	nd	nd	9.80 ± 0.84	6.41 ± 0.18	10.3 ± 0.3
	SA-GA	nd	nd	nd	22.01 ± 0.13	17.89 ± 0.45	28.8 ± 0.7
	SA-GEL	nd	nd	nd	11.50 ± 0.41	19.52 ± 0.10	31.5 ± 0.2
Syringic acid	CSE	9.94 ± 0.04	-	3.10 ± 0.06	-	nd	0.0
	SA	3.89 ± 0.09	3.84 ± 0.37	3.59 ± 0.15	nd	nd	0.0
	SA-GA	3.79 ± 0.00	4.53 ± 0.06	4.93 ± 0.49	nd	nd	0.0
	SA-GEL	4.57 ± 0.44	4.52 ± 0.62	4.76 ± 0.67	nd	nd	0.0
Vanillic acid	CSE	nd	-	nd	-	2.39 ± 0.11	22.9 ± 1.1
	SA	nd	nd	nd	3.86 ± 0.09	4.42 ± 0.62	42.3 ± 5.9
	SA-GA	nd	nd	nd	4.52 ± 0.06	5.59 ± 0.19	57.4 ± 1.9
	SA-GEL	nd	nd	nd	6.26 ± 0.82	7.40 ± 0.62	70.9 ± 5.9
Ellagic acid	CSE	nd	-	nd	-	1.63 ± 0.11	1.7 ± 0.1
	SA	nd	2.10 ± 0.07	2.18 ± 0.13	13.66 ± 0.31	12.67 ± 0.40	13.4 ± 0.4
	SA-GA	nd	nd	nd	2.37 ± 0.00	6.18 ± 0.06	6.5 ± 0.1
	SA-GEL	1.42 ± 0.52	3.21 ± 0.10	4.55 ± 0.87	30.85 ± 2.06	25.84 ± 0.62	27.3 ± 0.7

Table S1. – continued

Component	Sample	Oral phase OP$_3$	Gastric phase GP$_{63}$	Gastric phase GP$_{123}$	Intestinal phase IP$_{183}$	Intestinal phase IP$_{243}$	Bioaccessibility index *BI* (%)
***Phenolic acids* (µg/100 mg$_{EXT}$)**							
p-Hydroxybenzoic acid	CSE	nd	-	nd	-	nd	0.0
	SA	nd	nd	nd	nd	nd	0.0
	SA-GA	nd	nd	nd	nd	nd	0.0
	SA-GEL	nd	nd	nd	nd	nd	0.0
o-Coumaric acid	CSE	nd	-	nd	-	5.87 ± 0.93	78.7 ± 12.4
	SA	10.96 ± 0.41	21.80 ± 0.11	22.08 ± 0.24	16.53 ± 0.57	15.65 ± 0.31	209.8 ± 4.1
	SA-GA	nd	nd	nd	29.54 ± 0.45	26.35 ± 0.39	353.2 ± 5.2
	SA-GEL	15.91 ± 0.70	18.23 ± 0.62	30.29 ± 2.31	18.55 ± 1.34	15.46 ± 1.74	207.2 ± 23.4
p-Coumaric acid	CSE	0.59 ± 0.14	-	0.21 ± 0.07	-	2.99 ± 0.11	87.0 ± 3.3
	SA	nd	nd	nd	4.64 ± 0.04	0.68 ± 0.00	20.0 ± 0.0
	SA-GA	nd	nd	nd	nd	nd	0.0
	SA-GEL	nd	nd	nd	nd	nd	0.0
Caffeic acid	CSE	nd	-	nd	-	nd	0.0
	SA	nd	nd	nd	nd	nd	0.0
	SA-GA	nd	nd	nd	nd	nd	0.0
	SA-GEL	nd	nd	nd	nd	nd	0.0
Ferulic acid	CSE	nd	-	nd	-	nd	0.0
	SA	nd	nd	nd	nd	nd	0.0
	SA-GA	nd	nd	nd	nd	nd	0.0
	SA-GEL	nd	nd	nd	nd	nd	0.0
***Stilbenes* (µg/100 mg$_{EXT}$)**							
Resveratrol	CSE	nd	-	nd	-	nd	0.0
	SA	nd	nd	nd	nd	nd	0.0
	SA-GA	nd	nd	nd	nd	nd	0.0
	SA-GEL	nd	nd	nd	nd	nd	0.0
ε-Viniferin	CSE	nd	-	nd	-	nd	0.0
	SA	nd	nd	nd	nd	nd	0.0
	SA-GA	nd	nd	nd	nd	nd	0.0
	SA-GEL	nd	nd	nd	nd	nd	0.0

Table S1. – continued

Component	Sample	Oral phase	Gastric phase		Intestinal phase		Bioaccessibility index
		**OP_3**	**GP_{63}**	**GP_{123}**	**IP_{183}**	**IP_{243}**	***BI* (%)**
***Flavanols* (μg/100 mg_{EXT})**							
Epicatechin	CSE	79.11 ± 1.67	-	34.62 ± 0.71	-	50.19 ± 1.13	49.8 ± 1.1
	SA	47.93 ± 0.70	92.28 ± 0.84	87.55 ± 3.57	275.31 ± 1.06	261.25 ± 6.43	259.4 ± 6.4
	SA-GA	33.06 ± 0.06	78.24 ± 0.06	102.89 ± 7.62	297.23 ± 0.39	247.32 ± 8.80	245.6 ± 8.7
	SA-GEL	79.90 ± 1.08	101.49 ± 1.34	182.25 ± 17.90	287.26 ± 18.11	224.20 ± 0.10	222.6 ± 0.1
Catechin	CSE	61.78 ± 1.82	-	12.14 ± 0.57	-	nd	0.0
	SA	59.59 ± 0.64	55.82 ± 3.06	66.85 ± 1.28	nd	nd	0.0
	SA-GA	40.33 ± 0.02	56.31 ± 0.10	67.50 ± 6.64	nd	nd	0.0
	SA-GEL	54.37 ± 5.44	24.64 ± 4.74	74.91 ± 2.26	nd	nd	0.0
Epicatechin gallate	CSE	nd	-	6.99 ± 0.16	-	6.75 ± 0.76	116.8 ± 13.2
	SA	nd	24.12 ± 0.04	27.89 ± 0.75	26.61 ± 0.04	33.64 ± 2.86	582.6 ± 49.5
	SA-GA	nd	13.35 ± 0.13	15.59 ± 0.52	37.67 ± 0.32	38.11 ± 0.19	659.9 ± 3.4
	SA-GEL	nd	nd	6.69 ± 0.10	55.44 ± 6.59	55.38 ± 0.72	958.9 ± 12.4
Gallocatechin gallate	CSE	nd	-	nd	-	182.35 ± 7.51	251.4 ± 10.4
	SA	nd	nd	nd	333.26 ± 3.17	330.87 ± 8.58	456.2 ± 11.8
	SA-GA	nd	nd	nd	426.04 ± 0.58	478.95 ± 12.75	660.3 ± 17.6
	SA-GEL	nd	nd	nd	671.87 ± 19.14	716.07 ± 5.75	987.3 ± 7.9
Procyanidin B1	CSE	21.28 ± 1.36	-	nd	-	nd	0.0
	SA	26.27 ± 0.02	21.43 ± 3.50	18.75 ± 5.57	nd	nd	0.0
	SA-GA	nd	nd	nd	nd	nd	0.0
	SA-GEL	nd	nd	nd	nd	nd	0.0
Procyanidin B2	CSE	22.85 ± 0.34	-	8.81 ± 0.07	-	nd	0.0
	SA	14.38 ± 0.23	19.62 ± 1.17	18.24 ± 0.75	nd	nd	0.0
	SA-GA	7.98 ± 0.02	11.71 ± 0.06	15.68 ± 1.36	nd	nd	0.0
	SA-GEL	10.01 ± 0.90	nd	nd	nd	nd	0.0

Table S1. – continued

Component	Sample	Oral phase	Gastric phase		Intestinal phase		Bioaccessibility index
		OP_3	GP_{63}	GP_{123}	IP_{183}	IP_{243}	*BI* (%)
***Flavonols* (μg/100 mg$_{EXT}$)**							
Quercetin	CSE	17.75 ± 0.06	-	1.60 ± 0.08	-	nd	0.0
	SA	13.34 ± 0.18	17.60 ± 0.86	17.86 ± 0.31	nd	nd	0.0
	SA-GA	nd	nd	nd	nd	nd	0.0
	SA-GEL	7.70 ± 0.77	7.84 ± 1.50	11.67 ± 0.36	nd	nd	0.0
Rutin	CSE	4.95 ± 0.13	-	2.53 ± 0.07	-	nd	0.0
	SA	2.79 ± 0.53	3.25 ± 0.07	nd	nd	nd	0.0
	SA-GA	nd	nd	nd	nd	nd	0.0
	SA-GEL	nd	nd	nd	nd	nd	0.0
Kaempferol	CSE	nd	-	nd	-	nd	0.0
	SA	nd	nd	nd	nd	nd	0.0
	SA-GA	nd	nd	nd	nd	nd	0.0
	SA-GEL	nd	nd	nd	nd	nd	0.0
***Anthocyanins* (μg/100 mg$_{EXT}$)**							
Oenin chloride	CSE	47.12 ± 0.25	-	64.71 ± 0.01	-	31.42 ± 2.56	34.4 ± 2.8
	SA	28.57 ± 0.36	50.48 ± 0.35	46.26 ± 0.37	8.62 ± 0.13	7.91 ± 0.18	8.7 ± 0.2
	SA-GA	40.73 ± 3.87	15.23 ± 0.52	15.86 ± 0.26	nd	nd	0.0
	SA-GEL	57.80 ± 1.01	44.69 ± 1.96	44.03 ± 1.29	13.53 ± 0.62	12.92 ± 0.21	14.2 ± 0.2
Myrtillin chloride	CSE	2.29 ± 0.03	-	nd	-	nd	0.0
	SA	nd	nd	nd	nd	nd	0.0
	SA-GA	nd	nd	nd	nd	nd	0.0
	SA-GEL	nd	nd	nd	nd	nd	0.0
Petunidin chloride	CSE	0.96 ± 0.04	-	nd	-	nd	0.0
	SA	nd	nd	nd	nd	nd	0.0
	SA-GA	nd	nd	nd	nd	nd	0.0
	SA-GEL	nd	nd	nd	nd	nd	0.0
Peonidin-3-*O*-glucoside chloride	CSE	5.40 ± 0.04	-	6.29 ± 0.21	-	2.41 ± 0.42	24.7 ± 4.3
	SA	2.36 ± 0.21	4.39 ± 0.48	3.53 ± 0.24	nd	nd	0.0
	SA-GA	3.66 ± 0.34	1.81 ± 0.29	1.60 ± 0.13	nd	nd	0.0
	SA-GEL	4.08 ± 0.52	3.17 ± 0.77	2.65 ± 0.26	nd	nd	0.0

Table S1. – continued

Component	Sample	Oral phase	Gastric phase		Intestinal phase		Bioaccessibility index
		OP_3	GP_{63}	GP_{123}	IP_{183}	IP_{243}	*BI* (%)
Anthocyanins **(μg/100 mg$_{EXT}$)**							
Kuromanin chloride	CSE	nd	-	nd	-	nd	0.0
	SA	nd	nd	nd	nd	nd	0.0
	SA-GA	nd	nd	nd	nd	nd	0.0
	SA-GEL	nd	nd	nd	nd	nd	0.0

GP – gastric phase, IP – intestinal phase, OP – oral phase, nd – not detected, ″-„ – not determined. Index numbers associated with abbreviations indicate the time interval when a certain sample was taken (i.e. .GP_{123} – 123rd minute of the gastric phase). For the CSE, only the endpoints of the oral, gastric, and intestinal phases are shown (OP_3, GP_{123}, IP_{243}). Phenolic contents are expressed as mean value (μg/100 mg$_{EXT}$) ± SD.

Table S2. Content of individual phenolic compounds of phenol-rich grape pomace extract Cabernet Franc (CFE), sodium alginate microencapsulated powder (SA), sodium alginate with gum Arabic microencapsulated powder (SA-GA), and sodium alginate with gelatin microencapsulated powder (SA-GEL) during three phases of in vitro simulated digestion.

Component	Sample	Oral phase	Gastric phase		Intestinal phase		Bioaccessibility index
		OP_3	GP_{63}	GP_{123}	IP_{183}	IP_{243}	*BI* (%)
***Phenolic acids* (μg/100 mg_{EXT})**							
Gallic acid	CFE	2.09 ± 0.06	-	1.19 ± 0.17	-	141.38 ± 6.37	108.8 ± 4.9
	SA	0.86 ± 0.16	5.17 ± 0.96	3.16 ± 0.37	4.79 ± 0.00	16.01 ± 0.75	12.3 ± 0.6
	SA-GA	nd	6.15 ± 1.96	2.72 ± 0.51	102.85 ± 7.20	90.77 ± 1.22	69.8 ± 0.9
	SA-GEL	2.93 ± 0.08	8.47 ± 0.16	3.94 ± 0.11	11.65 ± 0.31	9.51 ± 0.00	7.3 ± 0.0
3,4-Dihydroxybenzoic acid	CFE	nd	-	nd	-	17.01 ± 0.08	70.6 ± 0.4
	SA	nd	nd	nd	13.95 ± 0.00	8.61 ± 0.04	35.7 ± 0.2
	SA-GA	nd	nd	nd	32.76 ± 1.99	30.00 ± 0.51	124.5 ± 2.1
	SA-GEL	nd	nd	nd	5.64 ± 0.21	15.90 ± 0.00	66.0 ± 0.0
Syringic acid	CFE	6.92 ± 0.78	-	nd	-	nd	0.0
	SA	3.49 ± 0.07	5.79 ± 0.09	3.75 ± 0.77	nd	nd	0.0
	SA-GA	9.51 ± 0.66	6.85 ± 0.51	8.92 ± 0.80	2.23 ± 0.19	2.36 ± 0.26	78.3 ± 8.5
	SA-GEL	nd	nd	nd	nd	nd	0.0
Vanillic acid	CFE	nd	-	nd	-	4.39 ± 0.00	29.6 ± 0.0
	SA	nd	nd	nd	3.92 ± 0.00	3.89 ± 0.13	26.2 ± 0.9
	SA-GA	nd	nd	nd	5.59 ± 0.58	5.23 ± 0.06	35.2 ± 0.4
	SA-GEL	nd	nd	nd	6.23 ± 0.00	6.98 ± 0.00	47.1 ± 0.0
Ellagic acid	CFE	nd	-	nd	-	1.28 ± 0.00	1.6 ± 0.0
	SA	nd	1.41 ± 0.15	1.73 ± 0.07	13.01 ± 0.00	13.46 ± 0.13	16.5 ± 0.2
	SA-GA	nd	nd	nd	9.54 ± 1.67	9.68 ± 0.45	11.9 ± 0.6
	SA-GEL	nd	1.57 ± 0.21	1.60 ± 0.05	16.77 ± 0.00	19.16 ± 0.00	23.6 ± 0.0

Table S2. – continued

Component	Sample	Oral phase OP$_3$	Gastric phase GP$_{63}$	Gastric phase GP$_{123}$	Intestinal phase IP$_{183}$	Intestinal phase IP$_{243}$	Bioaccessibility index *BI* (%)
***Phenolic acids* (µg/100 mg$_{EXT}$)**							
p-Hydroxybenzoic acid	CFE	nd	-	nd	-	nd	0.0
	SA	nd	nd	nd	nd	nd	0.0
	SA-GA	nd	nd	nd	nd	nd	0.0
	SA-GEL	nd	nd	nd	nd	nd	0.0
o-Coumaric acid	CFE	nd	-	nd	-	9.37 ± 0.06	48.1 ± 0.3
	SA	20.41 ± 0.20	27.06 ± 0.72	25.98 ± 0.57	18.49 ± 0.00	21.54 ± 0.66	110.6 ± 3.4
	SA-GA	nd	nd	nd	68.42 ± 5.78	82.63 ± 2.57	424.2 ± 13.2
	SA-GEL	17.23 ± 1.37	21.67 ± 1.21	22.92 ± 1.16	15.58 ± 0.00	19.76 ± 0.00	101.4 ± 0.0
p-Coumaric acid	CFE	0.43 ± 0.06	-	0.44 ± 0.00	-	3.93 ± 0.70	272.8 ± 49.0
	SA	nd	nd	nd	nd	nd	0.0
	SA-GA	nd	nd	nd	nd	nd	0.0
	SA-GEL	nd	nd	nd	nd	nd	0.0
Caffeic acid	CFE	nd	-	nd	-	nd	0.0
	SA	nd	nd	nd	nd	nd	0.0
	SA-GA	nd	nd	nd	nd	nd	0.0
	SA-GEL	nd	nd	nd	nd	nd	0.0
Ferulic acid	CFE	nd	-	nd	-	nd	0.0
	SA	nd	nd	nd	nd	nd	0.0
	SA-GA	nd	nd	nd	nd	nd	0.0
	SA-GEL	nd	nd	nd	nd	nd	0.0
***Stilbenes* (µg/100 mg$_{EXT}$)**							
Resveratrol	CFE	nd	-	nd	-	nd	0.0
	SA	nd	nd	nd	nd	nd	0.0
	SA-GA	nd	nd	nd	nd	nd	0.0
	SA-GEL	nd	nd	nd	nd	nd	0.0
ε-Viniferin	CFE	nd	-	nd	-	nd	0.0
	SA	nd	nd	nd	nd	nd	0.0
	SA-GA	nd	nd	nd	nd	nd	0.0
	SA-GEL	nd	nd	nd	nd	nd	0.0

Table S2. – continued

Component	Sample	Oral phase	Gastric phase		Intestinal phase		Bioaccessibility index
		OP_3	GP_{63}	GP_{123}	IP_{183}	IP_{243}	*BI* (%)
***Flavanols* (μg/100 mg$_{EXT}$)**							
Epicatechin	CFE	343.19 ± 7.06	-	324.16 ± 4.16	-	nd	0.0
	SA	160.11 ± 4.18	171.72 ± 0.64	180.02 ± 6.53	200.24 ± 0.00	167.33 ± 1.10	30.6 ± 0.2
	SA-GA	210.82 ± 0.90	352.28 ± 7.83	417.18 ± 4.40	100.63 ± 8.93	80.82 ± 2.31	14.8 ± 0.4
	SA-GEL	154.16 ± 4.14	185.78 ± 5.80	155.10 ± 1.26	107.82 ± 3.88	164.60 ± 1.68	30.1 ± 0.3
Catechin	CFE	100.95 ± 12.01	-	139.84 ± 2.90	-	nd	0.0
	SA	102.30 ± 2.23	77.12 ± 0.15	49.66 ± 3.21	nd	nd	0.0
	SA-GA	118.90 ± 0.08	96.28 ± 1.51	90.47 ± 8.70	nd	nd	0.0
	SA-GEL	21.09 ± 0.66	42.90 ± 1.37	45.21 ± 1.21	nd	nd	0.0
Epicatechin gallate	CFE	nd	-	nd	-	32.06 ± 0.06	98.8 ± 0.2
	SA	nd	nd	nd	27.46 ± 0.00	25.68 ± 1.06	79.1 ± 3.3
	SA-GA	nd	nd	nd	47.61 ± 3.34	51.82 ± 2.83	159.7 ± 8.7
	SA-GEL	nd	nd	nd	61.29 ± 2.52	54.82 ± 0.00	168.9 ± 0.0
Gallocatechin gallate	CFE	nd	-	nd	-	190.83 ± 6.03	150.0 ± 4.7
	SA	nd	nd	nd	381.74 ± 0.00	374.86 ± 5.85	294.6 ± 4.6
	SA-GA	nd	nd	nd	443.58 ± 6.17	450.08 ± 1.41	353.8 ± 1.1
	SA-GEL	nd	nd	nd	558.30 ± 1.68	601.79 ± 0.00	473.0 ± 0.0
Procyanidin B1	CFE	60.20 ± 0.10	-	72.36 ± 7.13	-	nd	0.0
	SA	81.43 ± 6.36	61.88 ± 2.52	49.69 ± 3.26	nd	nd	0.0
	SA-GA	68.20 ± 3.38	61.72 ± 4.24	66.34 ± 1.73	nd	nd	0.0
	SA-GEL	24.87 ± 4.01	nd	nd	nd	nd	0.0
Procyanidin B2	CFE	111.71 ± 5.15	-	137.25 ± 3.75	-	nd	0.0
	SA	3.49 ± 0.07	5.79 ± 0.09	3.75 ± 0.77	nd	nd	0.0
	SA-GA	64.41 ± 0.29	65.10 ± 4.08	92.86 ± 3.02	24.12 ± 4.95	34.95 ± 6.36	27.6 ± 5.0
	SA-GEL	31.92 ± 0.05	28.87 ± 0.95	38.66 ± 3.74	nd	nd	0.0

Table S2. – continued

Component	Sample	Oral phase	Gastric phase		Intestinal phase		Bioaccessibility index
		**OP_3**	**GP_{63}**	**GP_{123}**	**IP_{183}**	**IP_{243}**	***BI* (%)**
***Flavonols* (μg/100 mg_{EXT})**							
Quercetin	CFE	19.62 ± 0.67	-	15.89 ± 0.38	-	nd	0.0
	SA	nd	nd	nd	nd	nd	0.0
	SA-GA	nd	nd	nd	nd	nd	0.0
	SA-GEL	nd	nd	nd	nd	nd	0.0
Rutin	CFE	46.81 ± 0.68	-	64.19 ± 0.00	-	18.86 ± 1.80	29.0 ± 2.8
	SA	nd	nd	nd	nd	nd	0.0
	SA-GA	nd	nd	nd	nd	nd	0.0
	SA-GEL	nd	nd	nd	nd	nd	0.0
Kaempferol	CFE	nd	-	nd	-	nd	0.0
	SA	nd	nd	nd	nd	nd	0.0
	SA-GA	nd	nd	nd	nd	nd	0.0
	SA-GEL	nd	nd	nd	nd	nd	0.0
***Anthocyanins* (μg/100 mg_{EXT})**							
Oenin chloride	CFE	511.54 ± 1.17	-	733.77 ± 1.11	-	259.78 ± 23.24	32.7 ± 2.9
	SA	355.94 ± 2.52	224.77 ± 12.53	219.88 ± 33.29	61.27 ± 3.61	96.68 ± 10.81	12.2 ± 1.4
	SA-GA	378.04 ± 18.53	277.57 ± 2.05	306.86 ± 19.26	61.97 ± 1.28	65.32 ± 4.56	8.2 ± 0.6
	SA-GEL	310.17 ± 0.24	254.76 ± 0.58	248.77 ± 0.26	60.92 ± 0.94	49.10 ± 4.31	6.2 ± 0.5
Myrtillin chloride	CFE	7.98 ± 0.30	-	6.73 ± 0.01	-	nd	0.0
	SA	nd	nd	nd	nd	nd	0.0
	SA-GA	nd	nd	nd	nd	nd	0.0
	SA-GEL	nd	nd	nd	nd	nd	0.0
Petunidin chloride	CFE	3.11 ± 0.17	-	3.62 ± 0.22	-	nd	0.0
	SA	nd	nd	nd	nd	nd	0.0
	SA-GA	nd	nd	nd	nd	nd	0.0
	SA-GEL	nd	nd	nd	nd	nd	0.0
Peonidin-3-*O*-glucoside chloride	CFE	45.42 ± 0.90	-	61.59 ± 1.32	-	18.64 ± 2.23	24.0 ± 2.9
	SA	28.84 ± 1.23	16.32 ± 0.70	19.68 ± 2.71	4.36 ± 0.18	7.24 ± 0.31	9.3 ± 0.4
	SA-GA	29.92 ± 1.97	20.60 ± 0.06	22.77 ± 2.09	nd	nd	0.0
	SA-GEL	22.91 ± 1.61	19.44 ± 0.58	18.79 ± 0.58	nd	nd	0.0

Table S2. – continued

Component	Sample	Oral phase	Gastric phase		Intestinal phase		Bioaccessibility index
		**OP_3**	**GP_{63}**	**GP_{123}**	**IP_{183}**	**IP_{243}**	***BI* (%)**
***Anthocyanins* (μg/100 mg_{EXT})**							
Kuromanin chloride	CFE	nd	-	nd	-	nd	0.0
	SA	nd	nd	nd	nd	nd	0.0
	SA-GA	nd	nd	nd	nd	nd	0.0
	SA-GEL	nd	nd	nd	nd	nd	0.0
Callistephin chloride	CFE	nd	-	nd	-	nd	0.0
	SA	nd	nd	nd	nd	nd	0.0
	SA-GA	nd	nd	nd	nd	nd	0.0
	SA-GEL	nd	nd	nd	nd	nd	0.0

GP – gastric phase, IP – intestinal phase, OP – oral phase, nd – not detected, ˝-„ – not determined. Index numbers associated with abbreviations indicate the time interval when a certain sample was taken (i.e. GP_{123} – 123rd minute of the gastric phase). For the CFE, only the endpoints of the oral, gastric, and intestinal phases are shown (OP_3, GP_{123}, IP_{243}). Phenolic contents are expressed as mean value (μg/100 mg_{EXT}) ± SD.

Table S3. Content of individual phenolic compounds of phenol-rich grape pomace extract Merlot (ME), sodium alginate microencapsulated powder (SA), sodium alginate with gum Arabic microencapsulated powder (SA-GA), and sodium alginate with gelatin microencapsulated powder (SA-GEL) during three phases of in vitro simulated digestion.

**Component**	**Sample**	**Oral phase**	**Gastric phase**		**Intestinal phase**		**Bioaccessibility index**
		**OP_3**	**GP_{63}**	**GP_{123}**	**IP_{183}**	**IP_{243}**	***BI*** **(%)**
Phenolic acids **(μg/100 mg_{EXT})**							
Gallic acid	ME	1.57 ± 0.16	-	1.61 ± 0.04	-	98.41 ± 0.59	47.4 ± 0.3
	SA	nd	nd	nd	78.31 ± 9.37	30.06 ± 0.26	14.5 ± 0.1
	SA-GA	nd	nd	nd	113.77 ± 1.33	95.89 ± 0.00	46.2 ± 0.0
	SA-GEL	nd	nd	nd	101.35 ± 4.29	183.87 ± 0.61	88.5 ± 0.3
3,4-Dihydroxybenzoic acid	ME	nd	-	nd	-	9.33 ± 0.17	12.3 ± 0.2
	SA	nd	nd	nd	17.19 ± 0.60	8.59 ± 0.04	11.4 ± 0.1
	SA-GA	nd	nd	nd	21.94 ± 0.51	23.50 ± 0.00	31.1 ± 0.0
	SA-GEL	nd	nd	nd	24.18 ± 1.33	35.90 ± 0.51	47.5 ± 0.7
Syringic acid	ME	12.14 ± 2.90	-	11.88 ± 0.13	-	nd	0.0
	SA	3.49 ± 0.01	2.77 ± 0.19	4.09 ± 0.00	nd	nd	0.0
	SA-GA	7.04 ± 0.76	3.25 ± 0.60	nd	nd	nd	0.0
	SA-GEL	5.38 ± 0.03	nd	nd	nd	nd	0.0
Vanillic acid	ME	nd	-	nd	-	2.72 ± 0.08	22.1 ± 0.7
	SA	nd	nd	nd	3.80 ± 0.64	4.04 ± 0.30	32.7 ± 2.4
	SA-GA	nd	nd	nd	6.13 ± 0.19	6.52 ± 0.00	52.9 ± 0.0
	SA-GEL	nd	nd	nd	5.20 ± 0.20	6.55 ± 0.10	53.1 ± 0.8
Ellagic acid	ME	nd	-	nd	-	2.52 ± 0.08	15.5 ± 0.5
	SA	nd	nd	nd	2.37 ± 0.17	3.13 ± 0.04	19.2 ± 0.3
	SA-GA	nd	nd	nd	4.52 ± 0.19	6.12 ± 0.19	37.5 ± 1.2
	SA-GEL	nd	nd	nd	11.62 ± 1.33	13.16 ± 0.51	80.7 ± 3.1

Table S3. – continued

Component	Sample	Oral phase	Gastric phase		Intestinal phase		Bioaccessibility index
		**OP_3**	**GP_{63}**	**GP_{123}**	**IP_{183}**	**IP_{243}**	***BI* (%)**
***Phenolic acids* (μg/100 mg_{EXT})**							
p-Hydroxybenzoic acid	ME	nd	-	nd	-	nd	0.0
	SA	nd	nd	nd	nd	nd	0.0
	SA-GA	nd	nd	nd	nd	nd	0.0
	SA-GEL	nd	nd	nd	nd	nd	0.0
o-Coumaric acid	ME	nd	-	nd	-	5.92 ± 0.06	61.7 ± 0.6
	SA	nd	nd	nd	14.49 ± 0.13	15.49 ± 0.09	161.4 ± 0.9
	SA-GA	nd	nd	nd	25.57 ± 2.22	23.24 ± 0.00	242.2 ± 0.0
	SA-GEL	nd	nd	nd	28.87 ± 4.08	23.74 ± 2.44	247.4 ± 25.4
p-Coumaric acid	ME	0.28 ± 0.03	-	0.36 ± 0.03	-	2.16 ± 0.08	65.4 ± 2.5
	SA	nd	nd	nd	nd	nd	0.0
	SA-GA	nd	nd	nd	nd	nd	0.0
	SA-GEL	nd	nd	nd	nd	nd	0.0
Caffeic acid	ME	nd	-	nd	-	nd	0.0
	SA	nd	nd	nd	nd	nd	0.0
	SA-GA	nd	nd	nd	nd	nd	0.0
	SA-GEL	nd	nd	nd	nd	nd	0.0
Ferulic acid	ME	nd	-	nd	-	nd	0.0
	SA	nd	nd	nd	nd	nd	0.0
	SA-GA	nd	nd	nd	nd	nd	0.0
	SA-GEL	nd	nd	nd	nd	nd	0.0
***Stilbenes* (μg/100 mg_{EXT})**							
Resveratrol	ME	nd	-	nd	-	nd	0.0
	SA	nd	nd	nd	nd	nd	0.0
	SA-GA	nd	nd	nd	nd	nd	0.0
	SA-GEL	nd	nd	nd	nd	nd	0.0
ε-Viniferin	ME	nd	-	nd	-	nd	0.0
	SA	nd	nd	nd	nd	nd	0.0
	SA-GA	nd	nd	nd	nd	nd	0.0
	SA-GEL	nd	nd	nd	nd	nd	0.0

Table S3. – continued

Component	Sample	Oral phase	Gastric phase		Intestinal phase		Bioaccessibility index
		OP_3	GP_{63}	GP_{123}	IP_{183}	IP_{243}	*BI* (%)
Flavanols **(µg/100 mg_{EXT})**							
Epicatechin	ME	190.58 ± 9.18	-	200.57 ± 2.08	-	38.79 ± 0.53	15.6 ± 0.2
	SA	50.41 ± 0.00	181.44 ± 24.74	111.66 ± 0.00	219.09 ± 2.49	221.07 ± 3.99	89.0 ± 1.6
	SA-GA	138.47 ± 5.04	212.47 ± 20.05	167.31 ± 6.31	266.94 ± 11.40	227.80 ± 0.00	91.7 ± 0.0
	SA-GEL	103.49 ± 0.13	104.19 ± 0.00	179.46 ± 2.04	279.58 ± 8.88	223.65 ± 16.18	90.1 ± 6.5
Catechin	ME	101.90 ± 6.52	-	98.69 ± 1.03	-	nd	0.0
	SA	58.13 ± 0.10	52.31 ± 0.98	71.67 ± 0.00	nd	nd	0.0
	SA-GA	68.05 ± 3.73	52.45 ± 2.16	36.20 ± 5.81	nd	nd	0.0
	SA-GEL	62.14 ± 0.20	37.59 ± 0.00	73.99 ± 0.61	69.16 ± 11.03	56.83 ± 1.02	20.9 ± 0.4
Epicatechin gallate	ME	nd	-	nd	-	7.35 ± 0.73	88.7 ± 8.8
	SA	nd	nd	nd	26.40 ± 1.16	27.54 ± 1.07	332.4 ± 13.0
	SA-GA	nd	17.68 ± 1.46	17.79 ± 0.28	35.91 ± 4.69	39.32 ± 0.00	474.6 ± 0.0
	SA-GEL	nd	nd	nd	53.06 ± 3.57	59.13 ± 2.44	713.7 ± 29.5
Gallocatechin gallate	ME	104.18 ± 3.47	-	114.91 ± 2.29	-	178.11 ± 0.17	234.0 ± 0.2
	SA	nd	nd	nd	269.21 ± 1.46	277.94 ± 2.58	365.2 ± 3.4
	SA-GA	nd	nd	nd	405.34 ± 0.82	442.38 ± 0.00	581.2 ± 0.0
	SA-GEL	nd	nd	nd	747.57 ± 27.97	782.73 ± 7.63	1028.4 ± 10.0
Procyanidin B1	ME	28.71 ± 0.46	-	44.79 ± 5.83	-	nd	0.0
	SA	27.64 ± 0.00	27.82 ± 2.35	38.08 ± 0.00	nd	nd	0.0
	SA-GA	38.59 ± 1.25	27.73 ± 1.08	18.21 ± 3.28	nd	nd	0.0
	SA-GEL	nd	nd	nd	nd	nd	0.0
Procyanidin B2	ME	58.30 ± 2.27	-	61.28 ± 0.54	-	nd	0.0
	SA	14.74 ± 0.00	28.74 ± 0.19	19.83 ± 0.00	nd	nd	0.0
	SA-GA	40.40 ± 1.49	23.78 ± 2.03	21.67 ± 2.81	nd	nd	0.0
	SA-GEL	12.15 ± 0.13	55.77 ± 0.00	50.29 ± 0.10	nd	nd	0.0

Table S3. – continued

Component	Sample	Oral phase	Gastric phase		Intestinal phase		Bioaccessibility index
		**OP_3**	**GP_{63}**	**GP_{123}**	**IP_{183}**	**IP_{243}**	***BI* (%)**
***Flavonols* (µg/100 mg_{EXT})**							
Quercetin	ME	13.12 ± 0.81	-	5.93 ± 0.31	-	nd	0.0
	SA	nd	nd	nd	nd	nd	0.0
	SA-GA	nd	nd	nd	nd	nd	0.0
	SA-GEL	nd	nd	nd	nd	nd	0.0
Rutin	ME	7.00 ± 1.00	-	8.30 ± 0.01	-	nd	0.0
	SA	nd	nd	nd	nd	nd	0.0
	SA-GA	nd	nd	nd	nd	nd	0.0
	SA-GEL	nd	nd	nd	nd	nd	0.0
Kaempferol	ME	nd	-	nd	-	nd	0.0
	SA	nd	nd	nd	nd	nd	0.0
	SA-GA	nd	nd	nd	nd	nd	0.0
	SA-GEL	nd	nd	nd	nd	nd	0.0
***Anthocyanins* (µg/100 mg_{EXT})**							
Oenin chloride	ME	26.58 ± 0.64	-	38.08 ± 0.28	-	20.56 ± 1.49	62.6 ± 4.5
	SA	16.06 ± 1.29	13.09 ± 0.11	13.25 ± 0.00	9.27 ± 0.21	8.99 ± 0.09	27.4 ± 0.3
	SA-GA	12.66 ± 0.21	6.44 ± 0.22	5.33 ± 0.16	3.90 ± 0.44	2.86 ± 0.13	8.7 ± 0.4
	SA-GEL	14.26 ± 0.41	7.25 ± 0.46	9.11 ± 0.56	10.03 ± 0.10	8.06 ± 0.20	24.5 ± 0.6
Myrtillin chloride	ME	nd	-	nd	-	nd	0.0
	SA	nd	nd	nd	nd	nd	0.0
	SA-GA	nd	nd	nd	nd	nd	0.0
	SA-GEL	nd	nd	nd	nd	nd	0.0
Petunidin chloride	ME	0.27 ± 0.01	-	nd	-	nd	0.0
	SA	nd	nd	nd	nd	nd	0.0
	SA-GA	nd	nd	nd	nd	nd	0.0
	SA-GEL	nd	nd	nd	nd	nd	0.0
Peonidin-3-*O*-glucoside chloride	ME	5.49 ± 0.20	-	7.34 ± 0.13	-	3.04 ± 0.42	42.4 ± 5.9
	SA	2.69 ± 0.09	2.42 ± 0.17	2.17 ± 0.17	2.25 ± 0.17	1.61 ± 0.04	22.5 ± 0.6
	SA-GA	1.83 ± 0.05	nd	nd	nd	nd	0.0
	SA-GEL	2.14 ± 0.03	nd	nd	nd	nd	0.0

Table S3. – continued

Component	Sample	Oral phase	Gastric phase		Intestinal phase		Bioaccessibility index
		**OP_3**	**GP_{63}**	**GP_{123}**	**IP_{183}**	**IP_{243}**	***BI* (%)**
***Anthocyanins* (μg/100 mg_{EXT})**							
Kuromanin chloride	ME	nd	-	nd	-	nd	0.0
	SA	nd	nd	nd	nd	nd	0.0
	SA-GA	nd	nd	nd	nd	nd	0.0
	SA-GEL	nd	nd	nd	nd	nd	0.0

GP – gastric phase, IP – intestinal phase, OP – oral phase, nd – not detected, ˝-„ – not determined. Index numbers associated with abbreviations indicate the time interval when a certain sample was taken (i.e. GP_{123} – 123rd minute of the gastric phase). For the ME, only the endpoints of the oral, gastric, and intestinal phases are shown (OP_3, GP_{123}, IP_{243}). Phenolic contents are expressed as mean value (μg/100 mg_{EXT}) ± SD.
